# Nanomaterial-Based Strategies to Combat Antibiotic Resistance: Mechanisms and Applications

**DOI:** 10.3390/antibiotics14020207

**Published:** 2025-02-18

**Authors:** Nargish Parvin, Sang Woo Joo, Tapas K. Mandal

**Affiliations:** School of Mechanical Engineering, Yeungnam University, Gyeongsan 38541, Republic of Korea; nargish.parvin@gmail.com

**Keywords:** antibiotic resistance, nanomaterial-based antibacterial strategies, metal nanoparticles for infection control, nanotechnology in antibiotic delivery, combatting multidrug-resistant bacteria

## Abstract

The rapid rise of antibiotic resistance has become a global health crisis, necessitating the development of innovative strategies to combat multidrug-resistant (MDR) pathogens. Nanomaterials have emerged as promising tools in this fight, offering unique physicochemical properties that enhance antibiotic efficacy, overcome resistance mechanisms, and provide alternative therapeutic approaches. This review explores the diverse nanomaterial-based strategies used to combat antibiotic resistance, focusing on their mechanisms of action and practical applications. Nanomaterials such as metal nanoparticles, carbon-based nanomaterials, and polymeric nanostructures exhibit antibacterial properties through various pathways, including the generation of reactive oxygen species (ROS), disruption of bacterial membranes, and enhancement of antibiotic delivery. Additionally, the ability of nanomaterials to bypass traditional resistance mechanisms, such as biofilm formation and efflux pumps, has been demonstrated in numerous studies. This review also discusses the synergistic effects observed when nanomaterials are combined with conventional antibiotics, leading to increased bacterial susceptibility and reduced required dosages. By highlighting the recent advancements and clinical applications of nanomaterial–antibiotic combinations, this paper provides a comprehensive overview of how nanomaterials are reshaping the future of antibacterial therapies. Future research directions and challenges, including toxicity and scalability, are also addressed to guide the development of safer, more effective nanomaterial-based antibacterial treatments.

## 1. Introduction

### 1.1. Overview of the Global Antibiotic Resistance Crisis

The escalating crisis of antibiotic resistance has emerged as one of the most pressing challenges to global public health. Antibiotic resistance, defined as the ability of microorganisms to withstand the effects of antimicrobial agents, undermines decades of medical progress and threatens the effectiveness of existing therapies [1]. According to the World Health Organization (WHO), multidrug-resistant (MDR) pathogens are responsible for over 700,000 deaths annually, with projections suggesting that this number could rise to 10 million by 2050 if immediate action is not taken [2]. This phenomenon is driven by a combination of factors, including the misuse and overuse of antibiotics in clinical and agricultural settings, inadequate infection control measures, and the limited development of new antibiotics [3]. The situation is exacerbated by the rapid adaptability of bacteria, enabling them to develop sophisticated resistance mechanisms, such as the production of beta-lactamase enzymes, alteration of drug targets, and the activation of efflux pumps [4]. The economic burden of antibiotic resistance is equally alarming, with healthcare systems incurring significant costs due to prolonged hospital stays, additional diagnostic tests, and the need for more expensive and toxic treatments [5]. Beyond clinical settings, antibiotic resistance poses a significant threat to global food security, as resistant pathogens increasingly affect livestock and aquaculture industries [6]. Despite international initiatives, such as the Global Action Plan on Antimicrobial Resistance, there remains an urgent need for innovative solutions to combat this growing crisis [7].

### 1.2. Limitations of Conventional Antibiotics

Conventional antibiotics, once considered the cornerstone of modern medicine, are increasingly ineffective against resistant pathogens. Traditional antibiotics target specific bacterial processes, such as cell wall synthesis, protein production, or DNA replication. However, the selective pressure exerted by these drugs promotes the emergence and proliferation of resistant strains [8]. The phenomenon of horizontal gene transfer further exacerbates the spread of resistance genes among bacterial populations, rendering many first-line and even last-resort antibiotics obsolete [9]. Another significant limitation of conventional antibiotics is their inability to penetrate biofilms, i.e., complex microbial communities encased in a protective extracellular matrix. Biofilms, commonly associated with chronic infections, exhibit up to 1000-fold increased resistance to antibiotics compared to planktonic bacteria [10]. This resistance arises from limited antibiotic penetration, altered microenvironmental conditions within the biofilm, and the presence of persister cells that can withstand antimicrobial attack [11]. Additionally, the development pipeline for new antibiotics has slowed dramatically due to scientific, economic, and regulatory challenges. Pharmaceutical companies face high costs and lengthy timelines for antibiotic development, coupled with limited financial returns, as antibiotics are typically used sparingly to mitigate resistance [12]. This stagnation has created a void in the arsenal of effective antimicrobial agents, leaving healthcare providers with few options for treating resistant infections. The limitations of conventional antibiotics extend beyond resistance. The nonspecific nature of many antibiotics often results in off-target effects, including the disruption of beneficial microbiota. This can lead to secondary infections, such as Clostridioides difficile colitis, and contribute to broader ecological imbalances [13]. These challenges underscore the necessity of alternative therapeutic approaches to address the multifaceted nature of antibiotic resistance.

### 1.3. Emergence of Nanomaterials as Potential Solutions

Nanomaterials have emerged as a promising frontier in the fight against antibiotic resistance, offering innovative solutions that address the limitations of conventional therapies. Defined as materials with dimensions less than 100 nanometers, nanomaterials possess unique physicochemical properties, such as high surface area-to-volume ratios, tunable surface chemistries, and the ability to interact at the molecular level with biological systems [14]. These properties enable nanomaterials to enhance the efficacy of existing antibiotics, bypass resistance mechanisms, and introduce novel antibacterial modalities. One of the primary mechanisms by which nanomaterials combat bacterial infections is through the generation of reactive oxygen species (ROS). Metal-based nanoparticles, such as silver, zinc oxide, and titanium dioxide, induce oxidative stress within bacterial cells, leading to the damage of membranes, proteins, and DNA [15]. This mechanism is particularly advantageous against resistant strains, as it circumvents traditional antibiotic targets. Additionally, nanomaterials can disrupt bacterial membranes directly, as observed with cationic polymeric nanoparticles that interact electrostatically with negatively charged bacterial surfaces [16]. Nanomaterials also demonstrate significant potential in overcoming biofilm-associated resistance. For instance, nanoparticles functionalized with targeting ligands can penetrate the extracellular matrix and deliver antimicrobial agents directly to biofilm-embedded bacteria [17]. Furthermore, the use of nanomaterials as carriers for antibiotic delivery allows for enhanced drug stability, controlled release, and targeted therapy, reducing the required dosage and minimizing off-target effects [18]. The synergistic combination of nanomaterials with conventional antibiotics represents another promising strategy. Studies have shown that such combinations can restore the efficacy of antibiotics against resistant strains by enhancing drug uptake or inhibiting resistance pathways [19]. For example, gold nanoparticles have been reported to potentiate the activity of vancomycin against methicillin-resistant Staphylococcus aureus (MRSA) by facilitating its interaction with bacterial membranes [20]. Despite these advances, the application of nanomaterials in antimicrobial therapy is not without challenges. Concerns regarding the toxicity, environmental impact, and scalability of nanomaterial production must be addressed to ensure their safe and widespread use [21]. Nevertheless, ongoing research and technological innovations continue to expand the potential of nanomaterials as transformative tools in the battle against antibiotic resistance. So, the integration of nanomaterials into antimicrobial strategies represents a paradigm shift in addressing the limitations of conventional antibiotics. By leveraging their unique properties, nanomaterials offer a multifaceted approach to combatting resistant pathogens, with the potential to revolutionize the future of antibacterial therapy.

Table 1 provides a comparative overview that highlights the fundamental differences and advantages of nanomaterial-based antibacterial strategies over conventional antibiotics. Conventional antibiotics rely on specific mechanisms, such as targeting bacterial cell wall synthesis or protein production, which makes them highly effective initially but also predisposes them to resistance development due to bacterial adaptability and gene transfer. In contrast, nanomaterial-based strategies employ diverse and non-specific modes of action, such as reactive oxygen species (ROS) generation and membrane disruption, which are less likely to be thwarted by resistance mechanisms. Furthermore, while conventional antibiotics exhibit limited efficacy against biofilms due to poor penetration and altered bacterial states, nanomaterials demonstrate superior biofilm-targeting capabilities through their ability to traverse the extracellular matrix and deliver drugs effectively. Another notable distinction lies in therapeutic flexibility. Conventional antibiotics are constrained by their fixed chemical structures and single-functionality, whereas nanomaterials offer tunability in size, surface chemistry, and composition, enabling multifunctional applications such as targeted delivery and diagnostic integration [22,23,24,25,26]. However, challenges persist in both approaches. While conventional antibiotic development faces economic and regulatory hurdles, nanomaterials must overcome concerns related to toxicity, environmental impact, and large-scale manufacturing. Despite these challenges, the synergistic potential of combining nanomaterials with traditional antibiotics holds great promise, offering enhanced efficacy and reduced resistance emergence. This comparative analysis underscores the transformative potential of nanotechnology in addressing the limitations of conventional antimicrobial therapies while paving the way for innovative and sustainable solutions.

## 2. Mechanisms of Antibiotic Resistance

### 2.1. Common Mechanisms in Multidrug-Resistant (MDR) Bacteria

#### 2.1.1. Biofilm Formation

Biofilm formation is one of the most significant mechanisms by which bacteria evade antibiotic treatment. A biofilm is a structured microbial community encased in a self-produced extracellular polymeric substance (EPS), which provides both physical and chemical protection against antimicrobial agents. The EPS matrix acts as a diffusion barrier, limiting the penetration of antibiotics into the biofilm [27]. Furthermore, the microenvironment within biofilms, such as reduced oxygen levels and pH gradients, can render bacteria metabolically inactive, making them less susceptible to antibiotics that target actively growing cells [28]. Additionally, the presence of persister cells within biofilms further complicates treatment. These specialized cells exhibit a dormant state, allowing them to survive high concentrations of antibiotics and contribute to the recurrence of infections once treatment is ceased [29]. Biofilms are commonly associated with chronic infections, including those found in medical devices, such as catheters and prosthetics, and natural tissues, such as lungs in cystic fibrosis patients [30]. This underscores the need for therapeutic strategies capable of disrupting biofilm architecture and targeting embedded bacteria.

**Figure 1:**
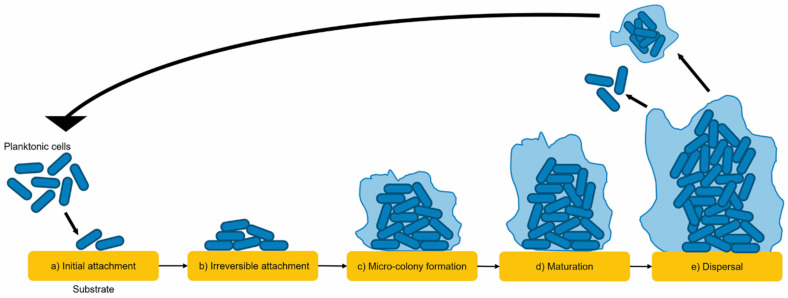
The development stages of a surface-associated biofilm. Reprinted/adapted with permission from Ref [31]. 2024, Ho Yu Liu et al.

Figure 1 depicted that biofilms are complex communities of microorganisms that adhere to surfaces and are embedded in a self-produced extracellular polymeric substance (EPS) matrix [31]. The lifecycle of a biofilm typically progresses through distinct stages: initial attachment, irreversible adhesion, maturation, and eventual dispersion. In the first stage, planktonic (free-floating) microbial cells encounter a surface and weakly adhere to it through van der Waals forces and hydrophobic interactions. This reversible attachment allows microorganisms to explore the surface for optimal adherence. Once conditions are favorable, irreversible adhesion occurs, facilitated by the microbial secretion of EPS, which acts as a glue anchoring cells to the surface. During the maturation phase, the biofilm structure becomes more complex as cells proliferate and produce additional EPS, creating a three-dimensional, highly organized matrix. This stage is characterized by the formation of microenvironments with gradients of nutrients, oxygen, and waste products, allowing the coexistence of diverse microbial populations with varying metabolic activities. Finally, in the dispersion phase, cells are released from the biofilm matrix back into the environment. This process is often triggered by nutrient depletion, quorum sensing signals, or environmental stress. The dispersed cells can colonize new surfaces, completing the biofilm lifecycle. Understanding these stages is critical for developing strategies to control biofilm formation, especially in clinical, industrial, and environmental contexts, where biofilms pose challenges such as antibiotic resistance and material degradation.

#### 2.1.2. Efflux Pumps

Efflux pumps are protein-based transport systems that actively expel antibiotics and other toxic compounds from bacterial cells, reducing the intracellular concentration of the drugs and rendering them ineffective. These pumps are encoded by genes that can be upregulated in response to antibiotic exposure, contributing to resistance against a broad spectrum of drugs [32]. Efflux pumps can be classified into several families, including the ATP-binding cassette (ABC) transporters, major facilitator superfamily (MFS), and resistance-nodulation-division (RND) family, each exhibiting varying substrate specificity and energy requirements [33]. For example, the AcrAB-TolC system in Escherichia coli and other Gram-negative bacteria has been extensively studied for its role in multidrug resistance [34]. Efflux pumps also play a critical role in biofilm-associated resistance, as the localized high expression of these systems within biofilms can further impede antibiotic efficacy [35]. Targeting efflux pump activity through inhibitors or designing antibiotics that bypass these systems presents a promising avenue for mitigating resistance [36].

#### 2.1.3. Enzymatic Degradation of Antibiotics

Enzymatic degradation represents a direct and effective mechanism by which bacteria neutralize antibiotics. Enzymes such as beta-lactamases cleave the beta-lactam ring of penicillins and cephalosporins, rendering these antibiotics ineffective [37]. Extended-spectrum beta-lactamases (ESBLs) and carbapenemases are particularly concerning due to their ability to degrade a wide range of beta-lactam antibiotics, including last-resort drugs like carbapenems [38]. Another example is the modification of aminoglycosides through acetylation, phosphorylation, or adenylation by aminoglycoside-modifying enzymes (AMEs), which significantly reduces the binding affinity of these antibiotics to their ribosomal targets [39]. The genetic determinants of these enzymes are often carried on mobile genetic elements, such as plasmids and transposons, facilitating their rapid dissemination among bacterial populations [40]. Overcoming enzymatic degradation requires innovative approaches, such as the use of enzyme inhibitors in combination with antibiotics or the development of molecules resistant to enzymatic modification [41].

## 3. Nanomaterials in Antibacterial Applications

The advent of nanotechnology has introduced a transformative paradigm in antibacterial applications, offering solutions to combat multidrug-resistant (MDR) pathogens and biofilm-associated infections. Nanomaterials, defined by their nanoscale dimensions (1–100 nm), exhibit unique physicochemical properties such as high surface area-to-volume ratios, tunable surface chemistry, and quantum effects, which collectively contribute to their potent antibacterial activity [42,43,44,45,46]. This section explores the types of nanomaterials commonly employed for antibacterial purposes, with a focus on metal-based and carbon-based nanomaterials.

### 3.1. Overview of Types of Nanomaterials Used for Antibacterial Purposes

#### 3.1.1. Metal-Based Nanoparticles

Metal-based nanoparticles (MNPs) are among the most extensively studied nanomaterials for antibacterial applications due to their broad-spectrum efficacy and multiple mechanisms of action.

- Silver Nanoparticles (AgNPs): Silver nanoparticles are one of the most widely used MNPs, renowned for their robust antibacterial properties. AgNPs exert their effects primarily through the generation of reactive oxygen species (ROS), which induce oxidative stress in bacterial cells. This results in the disruption of membranes, denaturation of proteins, and damage to DNA [47]. Additionally, silver ions released from AgNPs interact with thiol groups in bacterial enzymes, inhibiting metabolic processes [48]. AgNPs are particularly effective against biofilm-associated bacteria, as they can penetrate the extracellular matrix and target dormant cells [49]. However, concerns about their cytotoxicity and environmental impact necessitate further research to optimize their safe usage [50].
- Gold Nanoparticles (AuNPs): Gold nanoparticles are another class of MNPs with promising antibacterial properties. Although AuNPs are relatively inert, their functionalization with antimicrobial agents or ligands significantly enhances their efficacy. For instance, AuNPs conjugated with antibiotics, such as vancomycin, have demonstrated increased activity against methicillin-resistant Staphylococcus aureus (MRSA) by facilitating membrane interactions [20]. Additionally, AuNPs serve as excellent platforms for photothermal therapy, where they generate localized heat upon laser irradiation, effectively killing bacterial cells [51].
- Zinc Oxide Nanoparticles (ZnO NPs): Zinc oxide nanoparticles exhibit antibacterial activity through a combination of ROS generation, membrane disruption, and ionic interactions. ZnO NPs have been shown to damage bacterial membranes, leading to leakage of intracellular contents and cell death [52]. Their efficacy is further enhanced under UV light, which activates photocatalytic ROS production [53]. ZnO NPs are also biocompatible and degrade into non-toxic byproducts, making them attractive candidates for biomedical applications [54].
- Other Metal-Based Nanoparticles: Other MNPs, such as titanium dioxide (TiO_2_), copper oxide (CuO), and magnesium oxide (MgO), have also demonstrated antibacterial potential. TiO_2_ nanoparticles are particularly effective as photocatalysts, generating ROS under UV light to disrupt bacterial membranes and DNA [55]. CuO nanoparticles exhibit strong bactericidal effects against both Gram-positive and Gram-negative bacteria by disrupting membrane integrity and inhibiting enzyme activity [56].

#### 3.1.2. Carbon-Based Nanomaterials

Carbon-based nanomaterials (CBNs) are another class of nanomaterials with significant potential in antibacterial applications due to their unique structure, electrical properties, and biocompatibility.

- Graphene and Graphene Oxide (GO): Graphene and its derivative, graphene oxide (GO), exhibit strong antibacterial properties through multiple mechanisms. GO disrupts bacterial membranes via physical interactions, such as sharp-edge effects, leading to the leakage of cytoplasmic contents [57,58,59]. Additionally, GO induces oxidative stress and interferes with bacterial metabolic pathways. Functionalized graphene can also serve as a carrier for antibiotics, enhancing their stability and targeted delivery [60].
- Carbon Nanotubes (CNTs): Carbon nanotubes are cylindrical nanostructures composed of rolled graphene sheets. Their antibacterial activity arises from direct interactions with bacterial membranes, causing mechanical damage and oxidative stress [61]. CNTs can also penetrate biofilms, effectively targeting embedded bacteria [62]. Functionalized CNTs, conjugated with antimicrobial peptides or antibiotics, further enhance their therapeutic potential. However, concerns about their cytotoxicity and environmental persistence warrant further investigation.
- Fullerenes: Fullerenes, spherical carbon-based nanostructures, exhibit antibacterial activity primarily through ROS generation. Functionalized fullerenes, such as those conjugated with antimicrobial agents, show enhanced activity and biocompatibility [63].
- Other Carbon-Based Nanomaterials: Other CBNs, such as carbon dots and nanodiamonds, are emerging as promising antibacterial agents. Carbon dots, for example, exhibit strong ROS-generating capabilities and can be functionalized for targeted applications [64].

Both metal-based and carbon-based nanomaterials offer distinct advantages and challenges in antibacterial applications. While MNPs are highly effective against a broad range of bacteria, their potential cytotoxicity and environmental impact require careful consideration. On the other hand, CBNs, with their mechanical and oxidative mechanisms, provide a complementary approach to overcoming resistance but may face limitations in scalability and biocompatibility [65].

#### 3.1.3. Polymeric Nanostructures in Antibacterial Applications

Polymeric nanostructures have emerged as versatile platforms for antibacterial applications, offering tunable properties and multifunctionality. These materials are synthesized from polymers and designed in various architectures, such as dendrimers and nanogels, to achieve the targeted and controlled delivery of antimicrobial agents while minimizing toxicity. Their unique characteristics, such as biocompatibility, biodegradability, and the ability to encapsulate hydrophilic or hydrophobic drugs, make them valuable tools in combating multidrug-resistant (MDR) pathogens.

##### Nanogels

Nanogels are hydrophilic polymeric networks capable of encapsulating antimicrobial agents and responding to external stimuli, such as pH, temperature, or light. These properties make nanogels particularly suited for targeted antibacterial therapies. Nanogels act by delivering antimicrobial agents directly to bacterial cells, ensuring localized high concentrations of the drug. They can also be engineered to release their payload in response to environmental cues, such as the acidic pH of infected tissues [66]. In some cases, nanogels themselves exhibit antibacterial properties, especially when composed of cationic polymers that disrupt bacterial membranes [67]. Nanogels have shown significant potential in targeting biofilm-associated infections, where conventional antibiotics fail. For example, nanogels loaded with antibiotics or enzymes can penetrate the extracellular matrix of biofilms and eradicate embedded bacteria [68]. Their ability to respond to external stimuli further enhances their efficacy in challenging infection sites.

The advantages of nanogels include their high loading capacity, controlled drug release, and biocompatibility. However, challenges such as manufacturing complexity and potential instability in biological environments must be addressed to ensure their clinical translation.

### 3.2. Unique Physicochemical Properties of Nanomaterials That Enhance Antibacterial Activity

The remarkable antibacterial efficacy of nanomaterials arises from their unique physicochemical properties, which differentiate them from conventional antimicrobial agents. These properties enable nanomaterials to interact at the molecular and cellular levels with bacterial pathogens, overcoming traditional resistance mechanisms and enhancing therapeutic outcomes.

- High Surface Area-to-Volume Ratio: Nanomaterials possess an exceptionally high surface area-to-volume ratio, which enhances their interaction with bacterial cells. This property facilitates the attachment of nanomaterials to bacterial membranes, promoting membrane disruption, nutrient deprivation, and cellular damage [69]. For example, silver nanoparticles leverage their large surface area to release silver ions continuously, maintaining prolonged antibacterial activity [70].
- Size-Dependent Properties: The nanoscale dimensions of these materials enable them to penetrate bacterial cell walls and biofilms effectively. Nanoparticles smaller than 10 nm, for instance, can diffuse through the extracellular matrix of biofilms and reach bacterial cells that are otherwise inaccessible to conventional antibiotics [71]. This property is particularly useful in treating chronic infections associated with biofilms.
- Surface Functionalization and Tunability: The surface chemistry of nanomaterials can be tailored to enhance their antibacterial properties. Functionalization with cationic polymers, ligands, or antimicrobial peptides enables targeted interactions with bacterial cells while minimizing off-target effects. For example, gold nanoparticles functionalized with antibiotics such as vancomycin exhibit enhanced efficacy against resistant pathogens [72,73].
- Generation of Reactive Oxygen Species (ROS): Many nanomaterials, such as metal oxide nanoparticles, generate ROS that damage bacterial membranes, proteins, and DNA. Unlike conventional antibiotics, which target specific bacterial processes, ROS-mediated mechanisms are nonspecific and less prone to resistance development [74].
- Synergistic Effects with Antibiotics: Nanomaterials often exhibit synergistic effects when combined with conventional antibiotics. By enhancing drug uptake, preventing efflux, or disrupting resistance pathways, nanomaterials restore the efficacy of antibiotics against resistant strains. For example, graphene oxide combined with antibiotics has been shown to improve antibacterial activity by facilitating membrane penetration [75].
- Photothermal and Photodynamic Effects: Certain nanomaterials, such as gold nanoparticles and titanium dioxide, exhibit photothermal or photodynamic properties. Upon exposure to light, these materials generate localized heat or ROS, effectively killing bacterial cells without the need for traditional antibiotics [76].

Table 2 provides a comparative analysis of polymeric nanostructures, emphasizing their mechanisms of action, advantages, challenges, and practical applications in antibacterial therapies. Dendrimers, with their highly branched architecture, offer exceptional surface functionalization and molecular precision, enabling targeted delivery and membrane disruption. Their ability to interact electrostatically with bacterial membranes and generate reactive oxygen species (ROS) enhances their antibacterial efficacy. However, their therapeutic potential is constrained by cytotoxicity, complex synthesis, and potential immunogenic responses, particularly at higher concentrations. In contrast, nanogels are versatile due to their encapsulation capabilities, biodegradability, and stimulus-responsive behavior. Their ability to carry both hydrophilic and hydrophobic drugs makes them ideal for addressing complex infections, including biofilm-associated bacterial communities. Nonetheless, their limited mechanical stability under physiological conditions and scalability challenges remain significant barriers to widespread adoption. The examples highlighted in Table 2 illustrate how these nanostructures overcome traditional limitations of antibiotics. For instance, ciprofloxacin-loaded dendrimers demonstrate enhanced efficacy against multidrug-resistant (MDR) strains, while chitosan-based nanogels facilitate deeper penetration into biofilms, restoring antibiotic effectiveness. These findings underscore the transformative potential of polymeric nanostructures in developing next-generation antibacterial therapies. However, addressing the challenges of cytotoxicity, stability, and large-scale production is essential for translating these innovations into clinical applications.

## 4. Mechanisms of Action of Nanomaterials Against MDR Bacteria

### 4.1. Generation of Reactive Oxygen Species (ROS)

Nanomaterials are potent in combating multidrug-resistant (MDR) bacteria due to their ability to generate reactive oxygen species (ROS), a critical mechanism that induces bacterial cell damage. ROS are highly reactive molecules, including hydroxyl radicals (•OH), superoxide anions (O_2_^−^), and hydrogen peroxide (H_2_O_2_). These molecules cause oxidative stress in bacterial cells, leading to structural and functional damage that ultimately results in bacterial death.

#### 4.1.1. Role of Oxidative Stress in Bacterial Cell Damage

Oxidative stress arises when ROS generation surpasses the bacterial cell’s antioxidant defense mechanisms. This imbalance leads to the oxidation of essential biomolecules, disrupting cellular integrity and functionality:

- Damage to Lipid Membranes: Nanomaterials such as silver nanoparticles (AgNPs) and titanium dioxide nanoparticles (TiO_2_ NPs) induce ROS production, which targets the lipid bilayer of bacterial membranes. The oxidation of membrane lipids disrupts membrane integrity, causing permeability changes and leakage of intracellular components. This damage compromises critical bacterial processes, including ion transport and respiration, leading to cell lysis [77].
- Protein Oxidation: ROS generated by nanomaterials oxidize amino acid residues in bacterial proteins, particularly cysteine, methionine, and tyrosine. This leads to protein misfolding, enzymatic inactivation, and aggregation. For example, zinc oxide nanoparticles (ZnO NPs) have been shown to impair the bacterial enzymes involved in metabolic pathways, further exacerbating bacterial stress [78].
- DNA Damage: ROS can induce breaks in bacterial DNA strands and cause oxidative modifications, such as 8-hydroxydeoxyguanosine formation. These damages disrupt bacterial replication and transcription processes. Studies have demonstrated that graphene oxide nanoparticles (GO NPs) exhibit ROS-mediated antibacterial activity by inducing DNA fragmentation in MDR bacteria [79].

#### 4.1.2. Nanomaterial-Specific Contributions to ROS Generation

- Metal-Based Nanoparticles
  Metal-based nanoparticles, including AgNPs, TiO_2_ NPs, and ZnO NPs, are prominent ROS generators due to their redox activity and photocatalytic properties. For instance, TiO_2_ NPs produce ROS upon exposure to UV light, a property that has been effectively utilized in photodynamic antibacterial therapies [15,80].

- Carbon-Based Nanomaterials
  Graphene and its derivatives, such as graphene oxide and reduced graphene oxide, generate ROS through electron transfer reactions. These materials also physically damage bacterial membranes, enhancing their antibacterial efficacy [81,82].

- Polymeric Nanostructures
  Some polymeric nanostructures are designed to enhance ROS generation by incorporating ROS-generating agents or photosensitizers. These structures offer targeted antibacterial activity, minimizing damage to host cells [83].

The generation of ROS by nanomaterials provides a multifaceted mechanism that circumvents traditional bacterial resistance pathways, such as efflux pumps and enzymatic degradation. This makes ROS-based strategies particularly effective against MDR bacteria. However, challenges remain, including the following:

- Host Cytotoxicity: Excessive ROS levels can damage host tissues, necessitating precise control over ROS generation.
- Stability: The stability of ROS-generating nanomaterials under physiological conditions requires optimization for clinical applications.
- Environmental Impact: The long-term environmental effects of ROS-generating nanomaterials must be evaluated to ensure safety.

Hence, the ROS generation represents a robust and versatile mechanism for combating MDR bacteria. By inducing oxidative stress and targeting multiple bacterial components, nanomaterials hold significant promise for developing innovative antibacterial therapies. Ongoing research focuses on enhancing the specificity and safety of these strategies to ensure their clinical and environmental viability.

##### Dendrimers

Dendrimers are highly branched, tree-like polymeric nanostructures that exhibit a high degree of surface functionality and internal void space. These features allow dendrimers to serve as carriers for antimicrobial agents or act as standalone antibacterial agents.

- Mechanisms of Action: Dendrimers exert antibacterial activity primarily through membrane disruption and oxidative stress. Positively charged dendrimers, such as poly(amidoamine) (PAMAM), interact with negatively charged bacterial membranes, causing membrane destabilization and leakage of intracellular contents [84]. Additionally, dendrimers can generate reactive oxygen species (ROS), leading to oxidative damage and bacterial death.
- Drug Delivery and Synergistic Effects: Dendrimers are also employed as carriers for antibiotics, enhancing their solubility, stability, and bioavailability. For instance, ciprofloxacin-loaded PAMAM dendrimers have shown enhanced efficacy against Gram-negative bacteria compared to free ciprofloxacin [85]. Moreover, dendrimers can bypass resistance mechanisms by facilitating the intracellular delivery of antibiotics, thereby overcoming efflux pump-mediated resistance [86].

### 4.2. Disruption of Bacterial Membranes

#### How Nanomaterials Destabilize Bacterial Cell Walls

**Figure 2:**
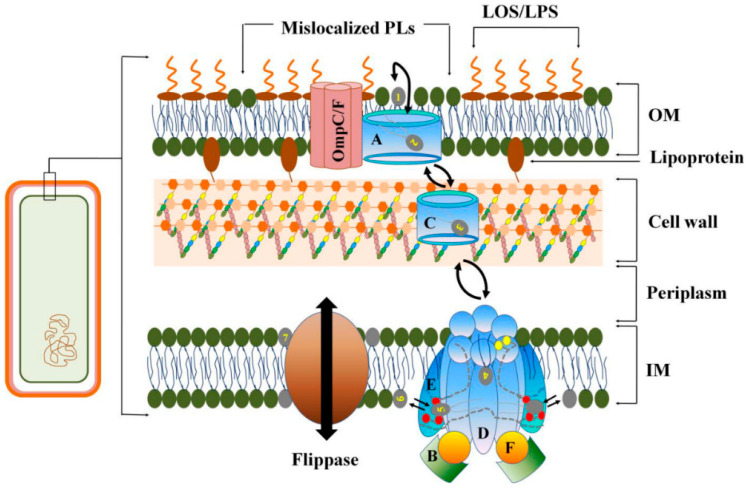
Role of the maintenance of lipid asymmetry (Mla) complex in the asymmetric distribution of LPSs and PLs in the outer and inner leaflets of a Gram-negative OM. In Gram-negative bacteria, the inner membrane (IM) consists of a symmetric phospholipid (PL) bilayer, whereas the outer membrane (OM) is asymmetric, with lipopolysaccharides (LPSs)/lipooligosaccharides (LOSs) exclusively occupying the outer leaflet and PLs confined to the inner leaflet. The maintenance of lipid asymmetry is orchestrated by the Mla complex, a six-component protein system in *Acinetobacter baumannii*, which is organized into three distinct units. The Mla complex consists of three main units: MlaA (A), MlaC (C), and MlaBDEF (B, D, E, and F). MlaA is positioned in the outer membrane (OM), MlaC is situated in the periplasm, and the MlaBDEF complex is located in the inner membrane (IM). The distinct structural properties of MlaA allow it to function similarly to a vacuum, extracting mislocalized phospholipids (PLs) from the outer leaflet of the outer membrane (OM). These mislocalized PLs are transferred to MlaC, which transports them to the PL binding site of the MlaBDEF complex, which faces the periplasm. The movement of PLs through the inner membrane (IM) occurs via the transmembrane domains of MlaD and MlaE, as shown by the dotted lines. The selective transport of PLs through the MlaBDEF complex is governed by specific residues: Leu153/Leu154 (yellow balls) at the periplasmic end, and R47, R14, and R234 (red balls) near the cytosolic end. ATP-binding sites are located on the MlaF subunits, near the interface with MlaE-F. The PL transport route within the MlaBDEF complex is bilaterally symmetrical, allowing PLs to pass through either path without preference. Once at the cytosolic side of MlaBDEF, the PLs move into the cytosolic leaflet of the IM, where they are balanced across both leaflets with the assistance of biogenic membrane flippases [87].

Nanomaterials exert potent antibacterial effects by disrupting bacterial membranes, targeting the structural integrity and protective function of the bacterial cell wall. Due to its unique composition and negatively charged surface, the bacterial membrane is particularly susceptible to nanomaterial interactions. The destabilization mechanisms vary based on the physicochemical properties of nanomaterials, including direct membrane penetration, oxidative stress induction, and lipid perturbation. One crucial pathway involved in maintaining membrane integrity in Gram-negative bacteria is the maintenance of lipid asymmetry (Mla) complex, which regulates the distribution of lipopolysaccharides (LPS) and phospholipids (PLs) between the outer and inner leaflets of the outer membrane (OM). Figure 2 highlights the Mla complex’s role in preserving the asymmetric lipid arrangement, where LPS localizes to the outer leaflet and PLs to the inner leaflet, while the inner membrane (IM) maintains a symmetric phospholipid bilayer. The Mla system, particularly in Acinetobacter baumannii, comprises six protein components, including MlaA, MlaC, and the MlaBDEF subcomplex, which collectively ensure proper PL localization. MlaA extracts mislocalized PLs at the OM and transfers them to MlaC, which shuttles them to the IM-located MlaBDEF complex. This complex then facilitates PL translocation across the IM via its transmembrane domains, forming a tightly regulated lipid transport pathway essential for membrane homeostasis. Nanomaterials, by interfering with this lipid transport system, can compromise membrane asymmetry and disrupt the selective permeability of bacterial membranes, leading to structural instability and increased susceptibility to antimicrobial agents. By directly targeting the Mla complex or indirectly perturbing lipid organization, nanomaterials enhance bacterial membrane fragility, ultimately contributing to their potent antibacterial activity.

Figure 2 illustrates the crucial role of the maintenance of lipid asymmetry (Mla) complex in maintaining the distinct distribution of lipopolysaccharides (LPSs) and phospholipids (PLs) between the outer and inner leaflets of the Gram-negative outer membrane (OM) [87]. In Gram-negative bacteria, the inner membrane (IM) is characterized by a symmetric bilayer of phospho-lipids, while the OM exhibits an asymmetric arrangement, with LPSs/LOSs localized exclusively to the outer leaflet and PLs confined to the inner leaflet. The Mla complex, particularly in Acinetobacter baumannii, is a six-component protein complex that includes MlaA, MlaC, and the MlaBDEF subcomplex. Each component plays a distinct role in ensuring the proper localization and maintenance of PLs. MlaA acts at the OM to extract mislocalized PLs, transferring them to MlaC, which then moves the PLs to the MlaBDEF complex located in the IM. The MlaBDEF complex facilitates the translocation of PLs across the IM through its transmembrane domains, forming a symmetrical pathway that permits the PLs to pass through either route. This system is essential for maintaining membrane integrity and asymmetry, and the unique structural features of the Mla complex allow for highly selective transport, ensuring that lipid distribution is tightly controlled to support cellular function.

- Electrostatic Interactions
  Many nanomaterials, such as cationic polymeric nanoparticles, possess positive surface charges. These charges electrostatically interact with the negatively charged lipopolysaccharides (LPSs) in Gram-negative bacteria or teichoic acids in Gram-positive bacteria. This interaction compromises membrane integrity, causing increased permeability, leakage of intracellular contents, and eventual bacterial death [88]. For instance, silver nanoparticles (AgNPs) effectively destabilize bacterial membranes through this mechanism, leading to rapid bacterial inactivation [89].

- Physical Disruption
  Nanomaterials such as graphene oxide (GO) and carbon nanotubes (CNTs) physically damage bacterial membranes. Their sharp edges or tubular structures insert into the lipid bilayer, creating pores and disrupting membrane continuity. This process leads to osmotic imbalance, loss of cytoplasmic content, and bacterial lysis [90].

- Lipid Peroxidation
  Reactive oxygen species (ROS) generated by nanomaterials induce lipid peroxidation in bacterial membranes. This oxidative modification weakens the membrane’s structural components, further destabilizing the cell wall. Metal oxide nanoparticles, such as zinc oxide (ZnO), are particularly effective in inducing lipid peroxidation, contributing to their strong antibacterial activity [52].

- Membrane Fusion and Fluidity Alteration
  Some nanomaterials alter the fluidity and phase behavior of bacterial membranes. Gold nanoparticles (AuNPs), functionalized with specific ligands, integrate into lipid bilayers, disrupting membrane dynamics and leading to functional impairment [91].

While membrane disruption is a highly effective antibacterial strategy, it poses challenges, such as potential damage to host cells and the need for precise targeting. To mitigate these risks, functionalization strategies are being developed to enhance bacterial specificity while reducing off-target effects.

### 4.3. Intracellular Delivery of Antibiotics

#### Nanoparticles as Carriers for the Targeted Delivery of Antibiotics

Nanoparticles serve as efficient carriers for the intracellular delivery of antibiotics, overcoming limitations of conventional antibiotic therapies, such as poor penetration into bacterial cells, biofilm-associated resistance, and systemic side effects.

- Enhanced Drug Stability and Solubility
  Nanoparticles improve the stability and solubility of antibiotics, protecting them from enzymatic degradation. For example, liposomes encapsulating vancomycin enhance its bioavailability and maintain its therapeutic efficacy in challenging environments [92,93].

- Targeted Delivery
  Functionalized nanoparticles can selectively target bacteria or infected tissues. Ligands, such as antibodies, peptides, or sugars, are conjugated to the nanoparticle surface to bind specifically to bacterial receptors. This approach ensures localized antibiotic release and minimizes damage to surrounding healthy cells [94].

- Controlled and Sustained Release
  Nanoparticles enable controlled and sustained drug release, ensuring therapeutic concentrations of antibiotics at the site of infection over extended periods. Polymeric nanoparticles, such as PLGA (poly(lactic-co-glycolic acid)), provide a tunable release profile, making them ideal for chronic infections [95].

- Overcoming Biofilm Resistance
  Nanoparticles penetrate the extracellular polymeric substance (EPS) of biofilms, delivering antibiotics directly to biofilm-embedded bacteria. For instance, chitosan nanoparticles loaded with ciprofloxacin have shown superior biofilm penetration and eradication capabilities compared to free ciprofloxacin [96].

- Combination Strategies
  Nanoparticles can co-deliver multiple therapeutic agents, such as antibiotics and adjuvants, to enhance antibacterial efficacy. For example, silver nanoparticles combined with antibiotics like tetracycline exhibit synergistic effects, restoring antibiotic sensitivity in resistant strains [97].

Nanoparticle-based antibiotic delivery offers precise targeting, reduced systemic toxicity, and improved treatment outcomes. However, challenges such as nanoparticle toxicity, immunogenicity, and scalability must be addressed. Current research focuses on developing biocompatible and biodegradable materials to overcome these hurdles and enable clinical translation.

### 4.4. Synergistic Effects with Traditional Antibiotics

#### 4.4.1. Advantages of Synergistic Combinations

The combination of nanomaterials with conventional antibiotics represents a promising strategy to combat multidrug-resistant (MDR) bacteria. This synergistic approach leverages the unique properties of nanomaterials to enhance the antibacterial potency of antibiotics, overcome resistance mechanisms, and reduce the required antibiotic dosage, mitigating associated side effects.

Nanomaterials facilitate the delivery and uptake of antibiotics into bacterial cells by disrupting the bacterial membrane or acting as carriers. For instance, silver nanoparticles (AgNPs) combined with ampicillin significantly enhance bacterial permeability, allowing greater antibiotic penetration and activity [98,99].

Many MDR bacteria form biofilms that act as protective barriers, preventing antibiotics from reaching the bacterial cells. Nanomaterials, such as graphene oxide (GO) and chitosan nanoparticles, disrupt the extracellular polymeric substance (EPS) of biofilms, exposing bacteria to the combined effects of antibiotics and nanomaterials. For example, a combination of GO and ciprofloxacin has shown remarkable efficacy in eradicating biofilm-embedded Pseudomonas aeruginosa [100].

The dual mechanisms of nanomaterials and antibiotics target bacteria simultaneously at multiple levels, reducing the likelihood of resistance development.

Nanoparticles like zinc oxide (ZnO) produce reactive oxygen species (ROS), which weaken bacterial defenses, enhancing the efficacy of antibiotics such as tetracycline. Nanomaterials inhibit bacterial enzymes (e.g., beta-lactamase) that degrade antibiotics, restoring the antibiotics’ effectiveness. Gold nanoparticles functionalized with beta-lactam antibiotics exemplify this synergy [101].

Efflux pumps are a common resistance mechanism in MDR bacteria, expelling antibiotics before they can act. Nanomaterials inhibit efflux pump activity, ensuring antibiotics remain within bacterial cells. For instance, carbon nanotubes have demonstrated the ability to block efflux pumps, enhancing the retention and efficacy of antibiotics like doxycycline [102].

By enhancing the activity of antibiotics, nanomaterial combinations reduce the required antibiotic dose, minimizing side effects and slowing the emergence of resistance. This approach is particularly beneficial for antibiotics with high systemic toxicity, such as aminoglycosides and polymyxins [103].

#### 4.4.2. Applications and Challenges of Synergistic Combinations

AgNPs combined with antibiotics like vancomycin or gentamicin exhibit superior antibacterial activity against both Gram-positive and Gram-negative MDR bacteria [104]. Graphene oxide functionalized with antibiotics such as levofloxacin demonstrates enhanced activity against drug-resistant strains of Escherichia coli and Staphylococcus aureus [105]. Chitosan nanoparticles loaded with antibiotics like rifampicin show enhanced penetration and effectiveness against biofilm-forming pathogens [106].

Table 3 provides a comparative overview of various mechanisms by which nanomaterials combat multidrug-resistant (MDR) bacteria, highlighting the different strategies that these materials employ to overcome bacterial resistance. One of the key mechanisms is the generation of reactive oxygen species (ROS), where nanomaterials like silver nanoparticles (AgNPs), zinc oxide nanoparticles (ZnO NPs), and graphene oxide (GO) produce ROS, causing oxidative stress and bacterial cell damage. This mechanism is particularly effective against MDR strains, as ROS can target a variety of bacterial biomolecules, reducing the potential for resistance development. However, controlling ROS levels is crucial to minimize cytotoxicity to host cells, and there are concerns about the environmental impact of ROS generation. Another important strategy is the disruption of bacterial membranes by nanomaterials like GO, carbon nanotubes (CNTs), and AgNPs. These materials physically interact with bacterial membranes, leading to structural damage, increased permeability, and leakage of intracellular components, offering a direct bactericidal action, especially against biofilm-forming bacteria. However, off-target effects on host cells and the need for bacterial specificity remain challenges in this approach. Nanomaterials also enhance intracellular antibiotic delivery, where materials such as liposomes, PLGA nanoparticles, and chitosan nanoparticles act as carriers, improving the stability, penetration, and localized delivery of antibiotics inside bacterial cells. This mechanism effectively overcomes biofilm resistance and reduces systemic toxicity, but issues like nanoparticle stability, immune clearance, and scalability need to be addressed. The synergistic effects with antibiotics represent another critical mechanism, where nanomaterials enhance the efficacy of conventional antibiotics by improving bacterial uptake, inhibiting efflux pumps, or disrupting biofilms. Combining AgNPs with antibiotics like ampicillin or GO with ciprofloxacin, for example, allows for reduced antibiotic doses, minimizes resistance, and enhances biofilm eradication. However, cytotoxicity concerns and ensuring stable interactions between nanomaterials and antibiotics are challenges that require careful consideration. Lastly, targeting specific bacterial mechanisms involves the inhibition of essential bacterial enzymes or disruption of metabolic pathways using gold nanoparticles (AuNPs) or functionalized graphene derivatives. This highly specific approach minimizes collateral damage to beneficial microbiota but comes with the challenges of high functionalization costs and the potential for bacterial adaptation to these targeted mechanisms. Overall, while nanomaterials hold great promise in combating MDR bacteria, overcoming the associated challenges will be key to their successful application in clinical settings.

## 5. Applications of Nanomaterials in Antibiotic Resistance

### 5.1. Nanomaterials as Antimicrobial Agents

Nanomaterials, including metal nanoparticles (e.g., silver, gold, and copper), carbon-based nanomaterials (such as graphene and carbon nanotubes), and polymeric nanostructures, exhibit significant antibacterial activity due to their unique physicochemical properties. The direct antibacterial effects of these nanoparticles are largely attributed to their high surface-area-to-volume ratio, which allows for enhanced interaction with bacterial cells. Metal nanoparticles, for instance, generate reactive oxygen species (ROS) upon interaction with bacteria, leading to oxidative stress, membrane damage, and cell death [107]. Additionally, the small size and large surface area of nanoparticles enable them to penetrate bacterial membranes more efficiently, disrupting cell wall synthesis and causing leakage of intracellular contents [108]. Carbon-based nanomaterials, such as graphene oxide, possess intrinsic antimicrobial properties that disrupt bacterial membranes via physical interactions and the formation of pores, facilitating the penetration of antibiotics or the release of toxic agents [109]. Moreover, the ability of nanomaterials to bypass bacterial resistance mechanisms, such as efflux pumps and biofilm formation, enhances their antimicrobial efficacy, making them an invaluable addition to the fight against multidrug-resistant (MDR) pathogens.

### 5.2. Nanocarriers for Enhanced Antibiotic Delivery

Nanocarriers have emerged as promising solutions for enhancing the delivery of antibiotics, improving their bioavailability, and overcoming challenges such as poor solubility and rapid clearance from the body. Nanocarriers, such as lipid nanoparticles, polymeric micelles, and dendrimers, can encapsulate antibiotics, protecting them from degradation and ensuring controlled release over time. The encapsulation of antibiotics in nanoparticles also enhances their stability, preventing premature degradation in physiological environments and prolonging their therapeutic effects [110]. Furthermore, nanocarriers facilitate the targeted delivery of antibiotics to infection sites, which increases local drug concentrations and minimizes systemic toxicity. For example, polymeric nanocarriers can be functionalized with targeting ligands to selectively bind to bacterial cell surfaces or infected tissues, improving the specificity and effectiveness of the antibiotic treatment [111]. The synergistic use of nanocarriers with antibiotics has also been shown to reduce the required dosage of antibiotics, thereby lowering the risk of developing antibiotic resistance. By improving the pharmacokinetic properties of antibiotics, nanocarriers offer a promising strategy to enhance the effectiveness of existing antibacterial agents and combat the growing challenge of MDR pathogens.

### 5.3. Nanomaterials Targeting Biofilm-Related Infections

Biofilms, which are aggregates of microbial cells encased in a self-produced extracellular matrix, present significant challenges in the treatment of infections, particularly those caused by multidrug-resistant (MDR) pathogens. Nanomaterials have emerged as effective agents for disrupting or preventing biofilm formation due to their unique surface characteristics, size, and ability to interact with bacterial cells. Metal nanoparticles, such as silver and copper nanoparticles, exhibit antimicrobial properties that can target the extracellular matrix of biofilms, disrupting the structural integrity and reducing the protective barrier against antibiotics [112]. Additionally, carbon-based nanomaterials like graphene oxide can penetrate biofilms and interact with bacterial cells, causing membrane damage and enhancing the permeability of antibiotics. Polymers and lipids can also be engineered to disrupt biofilm formation by interfering with the signaling pathways that mediate bacterial adhesion and aggregation, such as quorum sensing [113]. The combination of nanomaterials with conventional antibiotics has shown synergistic effects in biofilm eradication, reducing the necessary antibiotic concentrations and mitigating the development of resistance. These strategies offer promising solutions to tackle biofilm-associated infections, which are notoriously difficult to treat due to their inherent resistance to antibiotics and host immune responses.

### 5.4. Nanoparticles Overcoming Efflux Pump Mechanisms

Efflux pumps, which actively expel antibiotics from bacterial cells, are one of the major resistance mechanisms employed by MDR bacteria. Nanoparticles have shown great potential in overcoming this mechanism by enhancing the intracellular retention of antibiotics. Metallic nanoparticles, such as silver and gold, can inhibit the function of efflux pumps, preventing the expulsion of antibiotics and thereby increasing their intracellular concentration [114]. Carbon nanotubes and graphene oxide have been demonstrated to interact with bacterial cell membranes, facilitating the uptake of antibiotics and improving their intracellular retention [63]. Additionally, nanocarriers such as liposomes and micelles can encapsulate antibiotics and deliver them directly into the bacterial cell, bypassing the efflux pump mechanism. These nanoparticles can also be designed to selectively target bacterial cells, ensuring that the antibiotics remain within the infection site and are not rapidly expelled by bacterial resistance mechanisms. The combination of nanoparticles with antibiotics has been shown to restore antibiotic efficacy in resistant strains, reducing the dosage required and minimizing the risk of resistance development.

**Figure 3:**
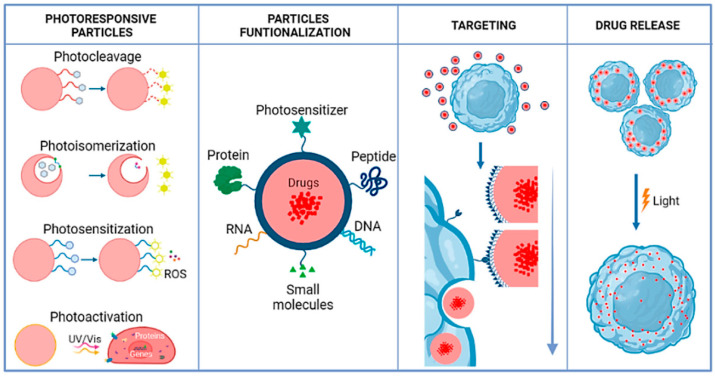
Diagrammatic representation of photosensitive polymeric nanocarriers: photosensitivity mechanisms, functionalized nanocarriers, targeted delivery, and controlled drug release [116].

Figure 3 illustrates the multifunctionality of photosensitive polymeric nanocarriers, which are designed to enhance precision in drug delivery systems. These nanocarriers exploit photosensitivity mechanisms, such as photothermal and photodynamic effects, triggered by specific wavelengths of light. Upon activation, these mechanisms facilitate structural changes in the polymer matrix, leading to controlled drug release. Functionalized nanocarriers, incorporating targeting ligands such as antibodies or peptides, enable specific interaction with diseased cells or tissues, minimizing off-target effects and improving therapeutic efficacy [115]. The targeting capability, combined with the external light-triggered release, allows for spatiotemporal control over drug delivery, reducing systemic toxicity and enhancing treatment outcomes. This schematic highlights the potential of these advanced nanocarriers for applications in precision medicine, particularly in treating localized diseases like cancer or infections. Despite their potential, dendrimers face challenges such as cytotoxicity at higher concentrations and complex synthesis procedures. Research is ongoing to optimize dendrimer designs for enhanced biocompatibility and scalability [116].

## 6. Current Challenges in the Use of Nanomaterials

### 6.1. Toxicity Concerns: Potential Risks Associated with Nanomaterials in Human Cells

While nanomaterials offer promising solutions in combating antibiotic resistance, their potential toxicity remains a significant concern. The unique physicochemical properties of nanomaterials, such as their small size, high surface area, and reactivity, can lead to unintended interactions with biological systems, posing risks to human cells. Nanoparticles may accumulate in tissues and organs, particularly in the liver, kidneys, and lungs, where they can induce oxidative stress, inflammation, and cell apoptosis [117]. The size and shape of nanoparticles influence their ability to penetrate cell membranes and accumulate within cells, potentially causing cytotoxicity. For example, metal nanoparticles like silver and copper can release toxic ions that disrupt cellular functions, leading to mitochondrial damage, DNA fragmentation, and cell death. Carbon-based nanomaterials, such as graphene oxide, have been shown to interact with cell membranes, leading to membrane rupture and cellular damage, while also eliciting inflammatory responses [118]. Furthermore, the long-term effects of nanomaterial exposure remain poorly understood, and the potential for bioaccumulation and chronic toxicity poses challenges for the widespread use of nanomaterials in clinical settings [119]. To mitigate these risks, it is essential to design and optimize nanomaterials with biocompatible coatings or surface modifications that reduce toxicity while maintaining their antimicrobial efficacy. Additionally, rigorous in vitro and in vivo testing, along with standardized safety protocols, are necessary to evaluate the safety profiles of nanomaterials before their clinical application.

### 6.2. Scalability and Manufacturing Issues: Challenges in the Large-Scale Production of Nanomaterials

Despite the potential of nanomaterials in combating antibiotic resistance, one of the major challenges hindering their widespread application is the scalability of production methods. The synthesis of nanomaterials on a small scale, under controlled laboratory conditions, often yields high-quality products with desired characteristics. However, translating these methods to large-scale production is complex and cost-prohibitive. One major issue is the difficulty in maintaining the uniformity and reproducibility of nanomaterial properties (e.g., size, shape, surface charge) at larger scales. Variations in these properties can influence the effectiveness and safety of nanomaterials in biomedical applications [120]. Moreover, conventional top–down and bottom–up methods, such as chemical vapor deposition (CVD), sol-gel synthesis, and laser ablation, can be energy-intensive and involve the use of toxic solvents, raising environmental and safety concerns. Additionally, the purification and functionalization processes required to optimize nanomaterials for therapeutic use can be laborious and costly, further complicating large-scale manufacturing [121]. To overcome these challenges, new, cost-effective, and environmentally sustainable methods need to be developed. Approaches like green synthesis using plant extracts or microbes, as well as scalable approaches such as microfluidics, are emerging as promising alternatives for large-scale production with minimal environmental impact [122]. Despite these advancements, achieving cost efficiency and maintaining quality control during large-scale production remain major obstacles to the commercialization of nanomaterials in the pharmaceutical and medical industries.

### 6.3. Regulatory Hurdles: Approval Pathways and Safety Testing

The introduction of nanomaterials into clinical applications is governed by stringent regulatory frameworks that ensure their safety and efficacy. However, regulatory bodies, such as the U.S. Food and Drug Administration (FDA) and the European Medicines Agency (EMA), face challenges in establishing guidelines that specifically address the unique properties of nanomaterials. Unlike traditional drugs or materials, nanomaterials exhibit unique physicochemical properties (such as size, surface area, and reactivity) that can significantly impact their behavior in biological systems. As a result, existing regulatory frameworks often fail to adequately assess the risks associated with the use of nanomaterials [123]. For example, conventional toxicity testing protocols may not be suitable for evaluating the potential risks of nanoparticles, which can behave differently from bulk materials. Thus, specialized methods for toxicity evaluation, including in vitro assays, animal models, and advanced imaging techniques, are essential for assessing the long-term effects of nanomaterial exposure. Furthermore, the regulatory approval process for nanomaterials is often lengthy and costly, involving extensive testing and documentation of safety profiles before clinical use. This can delay the translation of nanomaterial-based therapies from the laboratory to the clinic. Regulatory agencies are working toward establishing clearer guidelines for the development and approval of nanomaterials, but harmonization of global standards remains a challenge [124]. As the use of nanomaterials expands, addressing these regulatory hurdles and ensuring the safety of these materials for human use will be crucial for their successful integration into healthcare applications.

Table 4 provides a comparative overview of the current challenges in the use of nanomaterials, focusing on toxicity concerns, scalability and manufacturing issues, and regulatory hurdles. Toxicity remains a significant concern, as nanomaterials can interact with human cells in ways that traditional materials do not, potentially causing oxidative stress, inflammation, and cellular damage. These risks vary depending on the type of nanomaterial, such as metal nanoparticles or carbon-based materials, and necessitate specialized toxicity testing methods to evaluate safety. On the other hand, scalability and manufacturing issues highlight the challenges in achieving uniformity and reproducibility during large-scale production, with many traditional synthesis methods being energy-intensive or environmentally unfriendly. This impacts the cost effectiveness and sustainability of nanomaterial production, limiting their commercial viability. Furthermore, regulatory hurdles complicate the approval and clinical application of nanomaterials. Existing regulatory frameworks are not well equipped to assess the unique properties of nanomaterials, and specialized testing protocols are required to ensure safety. These challenges, including the lengthy approval process, contribute to delays in the commercialization of nanomaterial-based products. Addressing these issues through innovations in synthesis techniques, safety testing, and regulatory standards is essential to fully harness the potential of nanomaterials in healthcare and other industries.

## 7. Future Perspectives in Nanomaterial-Antibiotic Therapy

### 7.1. Advancements in Nanotechnology and Materials Science

Nanotechnology has witnessed significant advancements over the past few decades, particularly in the field of antibacterial therapy. The continuous evolution of nanomaterials has enabled the development of more effective strategies to combat multidrug-resistant (MDR) bacteria. Recent innovations in materials science have led to the synthesis of nanoparticles with tailored properties that enhance their therapeutic efficacy. Nanomaterials, such as metal nanoparticles (e.g., silver, gold, copper), carbon-based nanomaterials (e.g., graphene, carbon nanotubes), and polymeric nanoparticles, have shown excellent antibacterial properties due to their large surface area-to-volume ratio, which increases their interaction with bacterial cells. These nanoparticles exhibit multiple mechanisms of action, including membrane disruption, reactive oxygen species (ROS) generation, and enhanced antibiotic delivery, making them highly effective against resistant bacteria [125,126].

Recent advancements in nanomaterial functionalization have also contributed to improving the bioavailability and stability of antibiotics. For example, the encapsulation of antibiotics within nanocarriers like liposomes, micelles, and dendrimers allows for controlled and sustained release, overcoming the limitations of traditional antibiotic therapies. Moreover, the functionalization of nanoparticles with targeting ligands or antibodies enables site-specific drug delivery, reducing side effects and enhancing therapeutic outcomes [127]. Additionally, the integration of smart nanomaterials, such as pH-sensitive or temperature-responsive nanoparticles, allows for dynamic control over drug release in response to changes in the microenvironment, such as the acidic conditions commonly found at infection sites [128]. These innovations in nanomaterials are paving the way for more efficient and targeted antimicrobial therapies, which could significantly reduce the reliance on conventional antibiotics and combat the growing threat of antibiotic resistance.

### 7.2. Hybrid Nanomaterials for Enhanced Multifunctional Properties

Hybrid nanomaterials, which combine different nanomaterials or nanostructures, have emerged as a promising approach to enhance the multifunctional properties of antibacterial agents [129,130]. The combination of materials with complementary properties allows for the synergistic enhancement of antimicrobial effects, as well as the addition of other functionalities such as biofilm disruption, targeted delivery, and even therapeutic applications beyond antibacterial activity. For example, metal–organic frameworks (MOFs) and composite nanomaterials that combine metal nanoparticles with organic polymers or carbon-based materials have demonstrated superior antibacterial properties compared to individual nanomaterials. These hybrid materials can improve drug loading, enhance stability, and provide a sustained release of antibiotics, while also facilitating the prevention or disruption of bacterial biofilms, which are known to contribute to persistent infections [131]. In addition to antibacterial properties, hybrid nanomaterials can be designed to exhibit multiple functionalities, such as optical, magnetic, or electrochemical properties, enabling their use in diagnostic and imaging applications. For instance, gold nanoparticles combined with carbon nanotubes or graphene oxide have been used in the development of multifunctional platforms for bacterial detection and imaging, allowing for the simultaneous diagnosis and treatment of infections [131]. Furthermore, hybrid nanomaterials can be tailored to incorporate stimuli-responsive components, such as pH-sensitive polymers, that trigger drug release in response to changes in the local environment, ensuring that the therapeutic agents are delivered precisely to the infection site [128]. The versatility of hybrid nanomaterials opens up new avenues for designing advanced antibacterial therapies that can address multiple aspects of infection treatment, from diagnosis to targeted therapy, making them a valuable tool in the fight against antibiotic resistance.

### 7.3. Personalized Nanomedicine Approaches in Antibacterial Therapy

**Figure 4:**
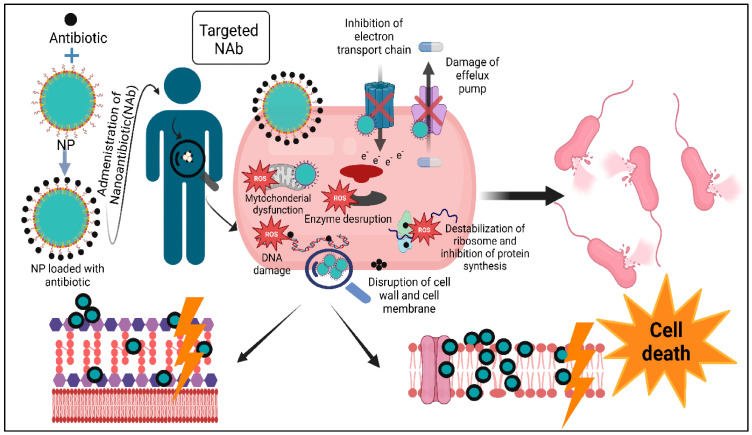
Mechanism of nanoparticle (NP)-mediated antibiotic delivery. NPs enhance the efficacy of conventional antibiotics (Ab) by targeting resistant microorganisms, providing dual mechanisms of action: the bactericidal effect of Ab and the antibacterial properties of NPs, leading to increased bacterial cell death. NPs disrupt the cell wall, membrane, mitochondria, and enzymes, and damage proteins, inhibit efflux pumps, and generate reactive oxygen species (ROS) to induce oxidative stress [135].

Personalized medicine is an emerging approach that tailors medical treatments to the individual characteristics of each patient, including genetic, environmental, and lifestyle factors. In the context of antibacterial therapy, personalized nanomedicine aims to optimize treatment by utilizing nanomaterials that are specifically designed to meet the unique needs of each patient, taking into account factors such as the bacterial strain involved, the patient’s immune system, and potential drug resistance patterns [132]. The use of nanomaterials in personalized medicine offers the potential to improve the effectiveness of antimicrobial treatments by targeting specific bacteria with high precision while minimizing adverse effects. One promising approach in personalized nanomedicine is the development of nanosystems that integrate diagnostic and therapeutic functions. For example, nanoparticles functionalized with specific ligands or antibodies can be used to selectively target bacterial strains based on their unique surface markers, ensuring that antibiotics are delivered directly to the site of infection. Additionally, the ability of nanoparticles to cross biological barriers, such as the blood–brain barrier, opens up new possibilities for treating infections in hard-to-reach areas of the body, such as the central nervous system [133]. Moreover, advances in nanotechnology have made it possible to design nanoparticles that can respond to the specific conditions of the patient’s body, such as the pH or temperature at the infection site, allowing for the controlled release of drugs tailored to the patient’s needs [134]. Personalized nanomedicine, through the use of custom-designed nanomaterials, promises to improve the success rates of antibacterial therapies by addressing individual variability in bacterial resistance and patient-specific factors. Figure 4 illustrates the dual mechanism of the action of nanoparticles (NPs) in delivering conventional antibiotics (Ab) to combat multidrug-resistant (MDR) bacteria [135]. NPs serve as carriers for antibiotics, enhancing their stability and facilitating targeted delivery to the bacterial cells. This approach not only boosts the antibacterial effectiveness of the antibiotic, but also leverages the inherent antibacterial properties of the NPs themselves. NPs can disrupt bacterial cell walls and membranes, leading to structural damage and cell lysis. Additionally, NPs induce mitochondrial damage, disrupt protein function, and interfere with essential cellular processes, such as the electron transport chain and enzyme activities. They also hinder the activity of efflux pumps, thereby preventing the removal of antibiotics from bacterial cells. Furthermore, NPs generate reactive oxygen species (ROS), which cause oxidative stress and damage critical cellular components like DNA, proteins, and lipids. This combination of antibiotic action and NP-mediated mechanisms results in potent bactericidal effects, making this strategy a promising approach for overcoming antibiotic resistance.

### 7.4. Potential for Clinical Translation and Real-World Applications

The clinical translation of nanomaterial-based antibacterial therapies holds significant promise for combating antibiotic-resistant infections. However, several challenges remain before these technologies can be widely adopted in clinical settings. One of the primary barriers to clinical translation is the need for extensive safety and toxicity testing, as the unique properties of nanomaterials can lead to unexpected interactions with biological systems. Despite the encouraging results observed in preclinical studies, the long-term effects of nanomaterial exposure on human health remain largely unknown, and more comprehensive studies are required to ensure their safety in vivo [136]. Another challenge is the scalability and reproducibility of nanomaterial synthesis. While laboratory-scale production of nanomaterials has shown great promise, large-scale production remains a significant hurdle due to concerns about cost, uniformity, and environmental impact. To address these issues, researchers are exploring greener and more cost-effective methods for producing nanomaterials at scale, such as using biological templates or environmentally benign solvents. Moreover, regulatory hurdles must be overcome before nanomaterial-based therapies can reach the market. Current regulatory frameworks are often ill-equipped to address the unique characteristics of nanomaterials, necessitating the development of new guidelines and testing protocols tailored to these innovative materials [137]. Despite these challenges, the potential applications of nanomaterial-based therapies in real-world settings are vast. Nanoparticles could be used not only to treat infections but also for the prevention of microbial colonization, biofilm formation, and chronic infections, all of which are difficult to manage with conventional antibiotics. In addition, nanomaterials could be integrated into medical devices, such as wound dressings or catheters, to provide continuous antimicrobial protection, reducing the risk of hospital-acquired infections. Furthermore, the combination of nanomaterials with existing antibiotics or other therapeutic modalities could lead to more effective treatment regimens that are less prone to resistance development [138,139,140,141]. As research in this field continues to advance, it is likely that nanomaterial-based antibacterial therapies will play an increasingly important role in the fight against antibiotic resistance and will eventually be integrated into clinical practice.

Table 5 highlights the diverse avenues through which nanomaterials are poised to transform antibacterial therapy. Advancements in nanotechnology and hybrid nanomaterials offer enhanced efficacy and multifunctionality, while personalized nanomedicine promises tailored treatments that increase precision and reduce side effects. The challenge lies in overcoming safety, scalability, and regulatory hurdles to enable the clinical translation of these technologies. As research progresses, nanomaterial-based therapies are expected to play a crucial role in combating the growing global threat of antibiotic resistance.

## 8. Conclusions

### 8.1. Summary of the Role of Nanomaterials in Combating Antibiotic Resistance

Nanomaterials have emerged as powerful tools in the fight against antibiotic resistance, offering unique properties that enhance the effectiveness of conventional antibiotics and introduce novel mechanisms of action. These materials, including metal nanoparticles, carbon-based nanomaterials, and polymeric nanostructures, exhibit antibacterial properties through various mechanisms such as the generation of reactive oxygen species, disruption of bacterial membranes, and enhancement of drug delivery. Additionally, their ability to bypass traditional resistance mechanisms like biofilm formation and efflux pumps positions them as a promising alternative to traditional antibiotics. Nanomaterials can also be engineered to overcome challenges such as the toxicity and bioavailability issues faced by many drugs, making them highly effective in combating multidrug-resistant (MDR) pathogens.

### 8.2. The Promise of Nanomaterial-Based Therapies in Addressing MDR Bacteria

Nanomaterial-based therapies hold immense promise in addressing the growing problem of multidrug-resistant bacteria. By combining the antimicrobial properties of nanomaterials with traditional antibiotics, synergistic effects can be achieved, reducing the required dosages and enhancing the overall therapeutic outcome. These advanced therapies not only target the bacterial cells directly but also offer solutions for tackling biofilm-related infections, which are often difficult to treat with conventional antibiotics. Furthermore, the use of nanomaterials in drug delivery systems ensures that antibiotics reach their target site more efficiently, reducing the likelihood of resistance development. As research in nanotechnology advances, these therapies could potentially revolutionize the treatment of chronic and hospital-acquired infections, improving the overall management of MDR bacteria.

### 8.3. Future Directions and Potential Impact on Global Health

Looking ahead, the future of nanomaterial-based therapies for antibiotic resistance is both exciting and full of potential. Continued advancements in nanotechnology will likely lead to the development of more effective, targeted, and personalized treatments that can be tailored to the specific needs of patients and pathogens. The integration of hybrid nanomaterials, which combine multiple functionalities, will open new avenues for multifunctional therapeutic strategies, including antimicrobial, diagnostic, and drug-delivery applications. Furthermore, personalized nanomedicine approaches could allow for more precise treatments, minimizing side effects and optimizing outcomes for individuals. Despite the challenges of toxicity, scalability, and regulatory hurdles, nanomaterials have the potential to play a pivotal role in addressing global health concerns related to antibiotic resistance. Their application could significantly reduce the burden of infectious diseases, improving public health outcomes worldwide and reshaping the landscape of antimicrobial therapy.

## Figures and Tables

**Table 1:** Comparative overview of conventional antibiotics and nanomaterial-based antibacterial strategies.

Aspect	Conventional Antibiotics	Nanomaterial-Based Strategies
Mechanism of Action	Targets specific bacterial processes (e.g., cell wall synthesis, protein production)	Induces oxidative stress, disrupts membranes, delivers antibiotics directly
Resistance Development	High, due to selective pressure and horizontal gene transfer	Lower, as mechanisms bypass traditional resistance pathways
Efficacy in Biofilms	Limited	High, due to penetration and targeted delivery
Therapeutic Flexibility	Low, fixed mechanisms	High, tunable properties and multifunctionality
Development Challenges	Slowed pipeline, high cost, and low financial returns	Scalability, toxicity, and regulatory concerns
Synergistic Potential	Limited	High, enhances efficacy of existing antibiotics

**Table 2:** Detailed comparative overview of polymeric nanostructures for antibacterial applications.

Type of Polymeric Nanostructure	Mechanisms of Action	Advantages	Challenges	Examples and Applications
Dendrimers	- Membrane disruption through electrostatic interactions - ROS generation - Drug delivery and release	- High degree of surface functionalization allows for targeted modification - Enhanced solubility and stability of encapsulated drugs - Precise molecular weight and size control enable predictable pharmacokinetics	- Cytotoxicity at high concentrations - Complex and costly synthesis - Potential immunogenicity	- PAMAM dendrimers with ciprofloxacin exhibit increased antibacterial efficacy against MDR *E. coli* and *S. aureus* [1].
Nanogels	- Encapsulation and controlled release of antibiotics - Biofilm penetration - Responsive behavior under stimuli (e.g., pH, temperature)	- Biodegradable and biocompatible materials - Ability to load both hydrophilic and hydrophobic drugs - Potential for multifunctional delivery with minimal off-target effects	- Limited mechanical stability under physiological conditions - Difficulties in scaling production	- Chitosan-based nanogels loaded with vancomycin effectively disrupt *S. aureus* biofilms by enhancing drug penetration [2].

**Table 3:** Comparative mechanisms of action of nanomaterials against MDR bacteria.

Mechanism	Description	Nanomaterial Examples	Advantages
Generation of ROS	Production of reactive oxygen species (e.g., hydroxyl radicals, hydrogen peroxide) causing oxidative stress and bacterial cell damage.	Silver nanoparticles (AgNPs), zinc oxide nanoparticles (ZnO NPs), graphene oxide (GO), titanium dioxide (TiO_2_ NPs).	Effective against MDR strains; targets multiple bacterial biomolecules; minimal resistance development.
Disruption of Membranes	Nanomaterials interact with bacterial membranes, leading to structural disruption, increased permeability, and leakage of intracellular components.	Graphene oxide (GO), carbon nanotubes (CNTs), silver nanoparticles (AgNPs), cationic polymeric nanoparticles.	Direct bactericidal action; effective against biofilm-forming bacteria.
Intracellular Antibiotic Delivery	Nanomaterials act as carriers, enhancing antibiotic stability, penetration, and localized delivery within bacterial cells.	Liposomes, PLGA nanoparticles, chitosan nanoparticles.	Overcomes biofilm resistance; reduces systemic toxicity; enables sustained release.
Synergistic Effects with Antibiotics	Nanomaterials enhance the efficacy of traditional antibiotics by increasing bacterial uptake, inhibiting efflux pumps, or disrupting biofilms.	AgNPs with ampicillin, GO with ciprofloxacin, chitosan nanoparticles with rifampicin.	Reduced antibiotic dose; minimizes resistance development; enhanced biofilm eradication.
Targeting Specific Mechanisms	Nanomaterials inhibit bacterial enzymes or disrupt metabolic pathways essential for survival.	Gold nanoparticles (AuNPs), functionalized graphene derivatives.	Highly specific action; minimizes collateral damage to microbiota.

**Table 4:** Comparative overview of the current challenges in the use of nanomaterials.

Challenges	Toxicity Concerns	Scalability and Manufacturing Issues	Regulatory Hurdles
Key Issue	Potential risks to human cells due to nanoparticle exposure	Difficulty in maintaining uniformity and quality in large-scale production	Lack of clear regulatory frameworks specific to nanomaterials
Impact on Human Health	Can cause oxidative stress, inflammation, and cellular damage	Variability in nanoparticle properties can affect safety and efficacy	Standard safety testing may not be suitable for nanomaterials
Nanomaterial Types Affected	Metal nanoparticles (silver, gold), carbon-based nanomaterials, and more	All types of nanomaterials, including metals, polymers, and carbon-based structures	All types of nanomaterials (e.g., nanoparticles, nanocarriers, etc.)
Challenges in Assessment	Need for specialized toxicity testing methods (e.g., in vitro, in vivo assays)	Challenges in achieving cost-efficiency and environmental sustainability	Establishment of proper testing and approval pathways for nanomaterials
Proposed Solutions	Biocompatible coatings, surface modifications, and comprehensive testing	Green synthesis, microfluidics, and sustainable manufacturing methods	Clearer regulatory guidelines and international standardization
Effect on Commercialization	Delays in clinical use due to safety concerns	High production costs, lack of scalability affect commercialization	Extended approval timelines and high costs for regulatory compliance

**Table 5:** Comparative overview of future perspectives in nanomaterial–antibiotic therapy.

Future Perspective	Advancements in Nanotechnology and Materials Science	Hybrid Nanomaterials for Enhanced Multifunctional Properties	Personalized Nanomedicine Approaches in Antibacterial Therapy	Potential for Clinical Translation and Real-World Applications
Key Focus	Development of novel nanomaterials with enhanced antimicrobial properties	Combination of different nanomaterials for multifunctional antibacterial effects	Tailored therapies based on individual patient characteristics	Transition from laboratory-scale findings to clinical practice
Impact on Antibacterial Therapy	Increases the effectiveness and specificity of nanomaterials against resistant bacteria	Synergistic properties enhance the antibacterial efficacy and biofilm disruption	Enables targeted, site-specific treatment, improving therapeutic outcomes	Facilitates real-world implementation, ensuring effectiveness in diverse patient populations
Technological Innovations	Surface modification of nanoparticles, controlled release mechanisms, and smart nanomaterials	Metal–organic frameworks (MOFs), composite nanoparticles combining organic and inorganic materials	Nanoparticles with targeted ligands or stimuli-responsive release systems	Development of cost-effective, scalable manufacturing processes for clinical use
Challenges	Safety concerns, bioavailability, stability in vivo	Synthesis complexity, scalability, and reproducibility of hybrid materials	High cost, patient variability in response to therapy	Regulatory hurdles, cost, toxicity testing, and large-scale production
Applications	Antimicrobial treatments, drug delivery, diagnostic applications	Antibacterial treatment, biofilm disruption, diagnostic imaging	Antibiotic targeting, enhanced drug delivery, treatment personalization	Hospital-acquired infection prevention, long-term chronic infection management
Prospects for the Future	Increased specificity, efficiency, and reduction of side effects	Multi-functional therapies for comprehensive infection management	More precise and effective treatments for resistant bacteria, tailored to the patient	Integration into clinical practice with improved patient outcomes and reduced antibiotic resistance

## References

[1] Mehrotra R., Cukor D., McCurry S.M., Rue T., Roumelioti M.-E., Heagerty P.J., Unruh M. (2024). Effectiveness of Existing Insomnia Therapies for Patients Undergoing Hemodialysis. Ann. Intern. Med..

[2] Ahmed S.K., Hussein S., Qurbani K., Ibrahim R.H., Fareeq A., Mahmood K.A., Mohamed M.G. (2024). Antimicrobial Resistance: Impacts, Challenges, and Future Prospects. J. Med. Surg. Public Health.

[3] Manyi-Loh C., Mamphweli S., Meyer E., Okoh A. (2018). Antibiotic Use in Agriculture and Its Consequential Resistance in Environmental Sources: Potential Public Health Implications. Molecules.

[4] Jhalora V., Bist R. (2024). A Comprehensive Review of Molecular Mechanisms Leading to the Emergence of Multidrug Resistance in Bacteria. Indian J. Microbiol..

[5] Baciu A.-P., Baciu C., Baciu G., Gurau G. (2024). The Burden of Antibiotic Resistance of the Main Microorganisms Causing Infections in Humans—Review of the Literature. J. Med. Life.

[6] Founou L.L., Founou R.C., Essack S.Y. (2021). Antimicrobial Resistance in the Farm-to-Plate Continuum: More Than a Food Safety Issue. Future Sci. OA.

[7] Talebi Bezmin Abadi A., Rizvanov A.A., Haertlé T., Blatt N.L. (2019). World Health Organization Report: Current Crisis of Antibiotic Resistance. Bionanoscience.

[8] Uddin T.M., Chakraborty A.J., Khusro A., Zidan B.R.M., Mitra S., Bin Emran T., Dhama K., Ripon M.K.H., Gajdács M., Sahibzada M.U.K. (2021). Antibiotic Resistance in Microbes: History, Mechanisms, Therapeutic Strategies and Future Prospects. J. Infect. Public Health.

[9] Amábile-Cuevas C.F., Lund-Zaina S. (2024). Non-Canonical Aspects of Antibiotics and Antibiotic Resistance. Antibiotics.

[10] Sharma S., Mohler J., Mahajan S.D., Schwartz S.A., Bruggemann L., Aalinkeel R. (2023). Microbial Biofilm: A Review on Formation, Infection, Antibiotic Resistance, Control Measures, and Innovative Treatment. Microorganisms.

[11] Uruén C., Chopo-Escuin G., Tommassen J., Mainar-Jaime R.C., Arenas J. (2020). Biofilms as Promoters of Bacterial Antibiotic Resistance and Tolerance. Antibiotics.

[12] Muteeb G., Rehman M.T., Shahwan M., Aatif M. (2023). Origin of Antibiotics and Antibiotic Resistance, and Their Impacts on Drug Development: A Narrative Review. Pharmaceuticals.

[13] Shim H. (2023). Three Innovations of Next-Generation Antibiotics: Evolvability, Specificity, and Non-Immunogenicity. Antibiotics.

[14] Khan S., Hossain M.K. (2022). Classification and Properties of Nanoparticles. Nanoparticle-Based Polymer Composites.

[15] Mammari N., Lamouroux E., Boudier A., Duval R.E. (2022). Current Knowledge on the Oxidative-Stress-Mediated Antimicrobial Properties of Metal-Based Nanoparticles. Microorganisms.

[16] Malekkhaiat Häffner S., Malmsten M. (2017). Membrane Interactions and Antimicrobial Effects of Inorganic Nanoparticles. Adv. Colloid Interface Sci..

[17] Zarepour A., Venkateswaran M.R., Khosravi A., Iravani S., Zarrabi A. (2024). Bioinspired Nanomaterials to Combat Microbial Biofilm and Pathogen Challenges: A Review. ACS Appl. Nano Mater..

[18] Nazli A., He D.L., Liao D., Khan M.Z.I., Huang C., He Y. (2022). Strategies and Progresses for Enhancing Targeted Antibiotic Delivery. Adv. Drug Deliv. Rev..

[19] León-Buitimea A., Garza-Cárdenas C.R., Román-García M.F., Ramírez-Díaz C.A., Ulloa-Ramírez M., Morones-Ramírez J.R. (2022). Nanomaterials-Based Combinatorial Therapy as a Strategy to Combat Antibiotic Resistance. Antibiotics.

[20] Sadeghi S., Agharazi F., Hosseinzadeh S.A., Mashayekhi M., Saffari Z., Shafiei M., Shahrokhi N., Ebrahimi-Rad M., Sadeghi M. (2024). Gold Nanoparticle Conjugation Enhances Berberine’s Antibacterial Activity against Methicillin-Resistant Staphylococcus Aureus (MRSA). Talanta.

[21] Ahmed S.F., Mofijur M., Rafa N., Chowdhury A.T., Chowdhury S., Nahrin M., Islam A.B.M.S., Ong H.C. (2022). Green Approaches in Synthesising Nanomaterials for Environmental Nanobioremediation: Technological Advancements, Applications, Benefits and Challenges. Environ. Res..

[22] Siwal S.S., Sheoran K., Mishra K., Kaur H., Saini A.K., Saini V., Vo D.-V.N., Nezhad H.Y., Thakur V.K. (2022). Novel Synthesis Methods and Applications of MXene-Based Nanomaterials (MBNs) for Hazardous Pollutants Degradation: Future Perspectives. Chemosphere.

[23] Kamble E., Sanghvi P., Pardesi K. (2022). Synergistic Effect of Antibiotic Combinations on Staphylococcus Aureus Biofilms and Their Persister Cell Populations. Biofilm.

[24] Coates A.R.M., Hu Y., Holt J., Yeh P. (2020). Antibiotic Combination Therapy against Resistant Bacterial Infections: Synergy, Rejuvenation and Resistance Reduction. Expert Rev. Anti-Infect. Ther..

[25] Cheesman M.J., Ilanko A., Blonk B., Cock I.E. (2017). Developing New Antimicrobial Therapies: Are Synergistic Combinations of Plant Extracts/Compounds with Conventional Antibiotics the Solution?. Pharmacogn. Rev..

[26] Zhu Y., Hao W., Wang X., Ouyang J., Deng X., Yu H., Wang Y. (2022). Antimicrobial Peptides, Conventional Antibiotics, and Their Synergistic Utility for the Treatment of Drug-Resistant Infections. Med. Res. Rev..

[27] Pinto R.M., Soares F.A., Reis S., Nunes C., Van Dijck P. (2020). Innovative Strategies Toward the Disassembly of the EPS Matrix in Bacterial Biofilms. Front. Microbiol..

[28] Bjarnsholt T., Whiteley M., Rumbaugh K.P., Stewart P.S., Jensen P.Ø., Frimodt-Møller N. (2022). The Importance of Understanding the Infectious Microenvironment. Lancet Infect. Dis..

[29] Huemer M., Mairpady Shambat S., Brugger S.D., Zinkernagel A.S. (2020). Antibiotic Resistance and Persistence—Implications for Human Health and Treatment Perspectives. EMBO Rep..

[30] Ciofu O., Tolker-Nielsen T., Jensen P.Ø., Wang H., Høiby N. (2015). Antimicrobial Resistance, Respiratory Tract Infections and Role of Biofilms in Lung Infections in Cystic Fibrosis Patients. Adv. Drug Deliv. Rev..

[31] Liu H.Y., Prentice E.L., Webber M.A. (2024). Mechanisms of Antimicrobial Resistance in Biofilms. npj Antimicrob. Resist..

[32] Elston R., Mulligan C., Thomas G.H. (2023). Flipping the Switch: Dynamic Modulation of Membrane Transporter Activity in Bacteria. Microbiology.

[33] Garcia Í.R., de Oliveira Garcia F.A., Pereira P.S., Coutinho H.D.M., Siyadatpanah A., Norouzi R., Wilairatana P., de Lourdes Pereira M., Nissapatorn V., Tintino S.R. (2022). Microbial Resistance: The Role of Efflux Pump Superfamilies and Their Respective Substrates. Life Sci..

[34] Alenazy R. (2022). Drug Efflux Pump Inhibitors: A Promising Approach to Counter Multidrug Resistance in Gram-Negative Pathogens by Targeting AcrB Protein from AcrAB-TolC Multidrug Efflux Pump from Escherichia Coli. Biology.

[35] Singh S., Datta S., Narayanan K.B., Rajnish K.N. (2021). Bacterial Exo-Polysaccharides in Biofilms: Role in Antimicrobial Resistance and Treatments. J. Genet. Eng. Biotechnol..

[36] Avakh A., Grant G.D., Cheesman M.J., Kalkundri T., Hall S. (2023). The Art of War with Pseudomonas Aeruginosa: Targeting Mex Efflux Pumps Directly to Strategically Enhance Antipseudomonal Drug Efficacy. Antibiotics.

[37] Alfei S., Schito A.M. (2022). β-Lactam Antibiotics and β-Lactamase Enzymes Inhibitors, Part 2: Our Limited Resources. Pharmaceuticals.

[38] Shaikh S., Fatima J., Shakil S., Rizvi S.M.D., Kamal M.A. (2015). Antibiotic Resistance and Extended Spectrum Beta-Lactamases: Types, Epidemiology and Treatment. Saudi J. Biol. Sci..

[39] El-Khoury C., Mansour E., Yuliandra Y., Lai F., Hawkins B.A., Du J.J., Sundberg E.J., Sluis-Cremer N., Hibbs D.E., Groundwater P.W. (2022). The Role of Adjuvants in Overcoming Antibacterial Resistance Due to Enzymatic Drug Modification. RSC Med. Chem..

[40] Frost L.S., Leplae R., Summers A.O., Toussaint A. (2005). Mobile Genetic Elements: The Agents of Open Source Evolution. Nat. Rev. Microbiol..

[41] Bilal M., Ashraf S.S., Barceló D., Iqbal H.M.N. (2019). Biocatalytic Degradation/Redefining “Removal” Fate of Pharmaceutically Active Compounds and Antibiotics in the Aquatic Environment. Sci. Total Environ..

[42] Hu X.-L., Shang Y., Yan K.-C., Sedgwick A.C., Gan H.-Q., Chen G.-R., He X.-P., James T.D., Chen D. (2021). Low-Dimensional Nanomaterials for Antibacterial Applications. J. Mater. Chem. B.

[43] Mandal T.K., Parvin N. (2011). Rapid Detection of Bacteria by Carbon Quantum Dots. J. Biomed. Nanotechnol..

[44] Parvin N., Mandal T.K. (2017). Dually Emissive P,N-Co-Doped Carbon Dots for Fluorescent and Photoacoustic Tissue Imaging in Living Mice. Microchim. Acta.

[45] Parvin N., Mandal T.K. (2016). Synthesis of a Highly Fluorescence Nitrogen-Doped Carbon Quantum Dots Bioimaging Probe and Its in Vivo Clearance and Printing Applications. RSC Adv..

[46] Parvin N., Kumar V., Manikkavel A., Park S.-S., Kumar Mandal T., Woo Joo S. (2023). Great New Generation Carbon Microsphere-Based Composites: Facile Synthesis, Properties and Their Application in Piezo-Electric Energy Harvesting. Appl. Surf. Sci..

[47] Roy A., Bulut O., Some S., Mandal A.K., Yilmaz M.D. (2019). Green Synthesis of Silver Nanoparticles: Biomolecule-Nanoparticle Organizations Targeting Antimicrobial Activity. RSC Adv..

[48] Jiang H.S., Zhang Y., Lu Z.W., Lebrun R., Gontero B., Li W. (2019). Interaction between Silver Nanoparticles and Two Dehydrogenases: Role of Thiol Groups. Small.

[49] Al-Wrafy F.A., Al-Gheethi A.A., Ponnusamy S.K., Noman E.A., Fattah S.A. (2022). Nanoparticles Approach to Eradicate Bacterial Biofilm-Related Infections: A Critical Review. Chemosphere.

[50] Saifi M.A., Khan W., Godugu C. (2018). Cytotoxicity of Nanomaterials: Using Nanotoxicology to Address the Safety Concerns of Nanoparticles. Pharm. Nanotechnol..

[51] Zou Y., Zhang Y., Yu Q., Chen H. (2021). Photothermal Bactericidal Surfaces: Killing Bacteria Using Light Instead of Biocides. Biomater. Sci..

[52] Sirelkhatim A., Mahmud S., Seeni A., Kaus N.H.M., Ann L.C., Bakhori S.K.M., Hasan H., Mohamad D. (2015). Review on Zinc Oxide Nanoparticles: Antibacterial Activity and Toxicity Mechanism. Nano-Micro Lett..

[53] Ma H., Brennan A., Diamond S.A. (2012). Photocatalytic Reactive Oxygen Species Production and Phototoxicity of Titanium Dioxide Nanoparticles Are Dependent on the Solar Ultraviolet Radiation Spectrum. Environ. Toxicol. Chem..

[54] Raha S., Ahmaruzzaman M. (2022). ZnO Nanostructured Materials and Their Potential Applications: Progress, Challenges and Perspectives. Nanoscale Adv..

[55] Yong S.-S., Lee J.-I., Kang D.-H. (2023). TiO2-Based Photocatalyst Generated Reactive Oxygen Species Cause Cell Membrane Disruption of Staphylococcus Aureus and Escherichia Coli O157:H7. Food Microbiol..

[56] Guan G., Zhang L., Zhu J., Wu H., Li W., Sun Q. (2021). Antibacterial Properties and Mechanism of Biopolymer-Based Films Functionalized by CuO/ZnO Nanoparticles against Escherichia Coli and Staphylococcus Aureus. J. Hazard. Mater..

[57] Mohammed H., Kumar A., Bekyarova E., Al-Hadeethi Y., Zhang X., Chen M., Ansari M.S., Cochis A., Rimondini L. (2020). Antimicrobial Mechanisms and Effectiveness of Graphene and Graphene-Functionalized Biomaterials. A Scope Review. Front. Bioeng. Biotechnol..

[58] Parvin N., Kumar V., Joo S.W., Park S.-S., Mandal T.K. (2022). Recent Advances in the Characterized Identification of Mono-to-Multi-Layer Graphene and Its Biomedical Applications: A Review. Electronics.

[59] Mandal T.K., Lee Y.R., Parvin N. (2020). Red Phosphorus Decorated Graphene Oxide Nanosheets: Label-Free DNA Detection. Biomater. Sci..

[60] Karki N., Tiwari H., Tewari C., Rana A., Pandey N., Basak S., Sahoo N.G. (2020). Functionalized Graphene Oxide as a Vehicle for Targeted Drug Delivery and Bioimaging Applications. J. Mater. Chem. B.

[61] Asaftei M., Lucidi M., Cirtoaje C., Holban A.-M., Charitidis C.A., Yang F., Wu A., Stanciu G.A., Sağlam Ö., Lazar V. (2023). Fighting Bacterial Pathogens with Carbon Nanotubes: Focused Review of Recent Progress. RSC Adv..

[62] Cui F., Li T., Wang D., Yi S., Li J., Li X. (2022). Recent Advances in Carbon-Based Nanomaterials for Combating Bacterial Biofilm-Associated Infections. J. Hazard. Mater..

[63] Azizi-Lalabadi M., Hashemi H., Feng J., Jafari S.M. (2020). Carbon Nanomaterials against Pathogens; the Antimicrobial Activity of Carbon Nanotubes, Graphene/Graphene Oxide, Fullerenes, and Their Nanocomposites. Adv. Colloid Interface Sci..

[64] Patel K.D., Keskin-Erdogan Z., Sawadkar P., Nik Sharifulden N.S.A., Shannon M.R., Patel M., Silva L.B., Patel R., Chau D.Y.S., Knowles J.C. (2024). Oxidative Stress Modulating Nanomaterials and Their Biochemical Roles in Nanomedicine. Nanoscale Horiz..

[65] Solano-Orrala D., Silva-Cullishpuma D.A., Díaz-Cruces E., Gómez-López V.M., Toro-Mendoza J., Gomez d’Ayala G., Troconis J., Narváez-Muñoz C., Alexis F., Mercader-Ros M.T. (2024). Exploring the Potential of Nonpsychoactive Cannabinoids in the Development of Materials for Biomedical and Sports Applications. ACS Appl. Bio Mater..

[66] Sivaram A.J., Rajitha P., Maya S., Jayakumar R., Sabitha M. (2015). Nanogels for Delivery, Imaging and Therapy. WIREs Nanomed. Nanobiotechnol..

[67] Keskin D., Zu G., Forson A.M., Tromp L., Sjollema J., van Rijn P. (2021). Nanogels: A Novel Approach in Antimicrobial Delivery Systems and Antimicrobial Coatings. Bioact. Mater..

[68] Ali A.A., Al Bostami R.D., Al-Othman A. (2024). Nanogel-Based Composites for Bacterial Antibiofilm Activity: Advances, Challenges, and Prospects. RSC Adv..

[69] He J., Hong M., Xie W., Chen Z., Chen D., Xie S. (2022). Progress and Prospects of Nanomaterials against Resistant Bacteria. J. Control. Release.

[70] Jangid H., Singh S., Kashyap P., Singh A., Kumar G. (2024). Advancing Biomedical Applications: An in-Depth Analysis of Silver Nanoparticles in Antimicrobial, Anticancer, and Wound Healing Roles. Front. Pharmacol..

[71] Malaekeh-Nikouei B., Fazly Bazzaz B.S., Mirhadi E., Tajani A.S., Khameneh B. (2020). The Role of Nanotechnology in Combating Biofilm-Based Antibiotic Resistance. J. Drug Deliv. Sci. Technol..

[72] Kuo Y.-L., Wang S.-G., Wu C.-Y., Lee K.-C., Jao C.-J., Chou S.-H., Chen Y.-C. (2016). Functional Gold Nanoparticle-Based Antibacterial Agents for Nosocomial and Antibiotic-Resistant Bacteria. Nanomedicine.

[73] Al Hagbani T., Yadav H., Moin A., Lila A.S.A., Mehmood K., Alshammari F., Khan S., Khafagy E.-S., Hussain T., Rizvi S.M.D. (2022). Enhancement of Vancomycin Potential against Pathogenic Bacterial Strains via Gold Nano-Formulations: A Nano-Antibiotic Approach. Materials.

[74] Dharmaraja A.T. (2017). Role of Reactive Oxygen Species (ROS) in Therapeutics and Drug Resistance in Cancer and Bacteria. J. Med. Chem..

[75] Xiao S., Lu X., Gou L., Li J., Ma Y., Liu J., Yang K., Yuan B. (2019). Graphene Oxide as Antibacterial Sensitizer: Mechanically Disturbed Cell Membrane for Enhanced Poration Efficiency of Melittin. Carbon.

[76] Wang Y., Yang Y., Shi Y., Song H., Yu C. (2020). Antibiotic-Free Antibacterial Strategies Enabled by Nanomaterials: Progress and Perspectives. Adv. Mater..

[77] Tigabu B., Getachew A. (2022). Treatment of Antibiotic-Resistant Bacteria by Nanoparticles: Current Approaches and Prospects. Ann. Adv. Chem..

[78] Cameron S.J., Sheng J., Hosseinian F., Willmore W.G. (2022). Nanoparticle Effects on Stress Response Pathways and Nanoparticle–Protein Interactions. Int. J. Mol. Sci..

[79] Cavallo D., Chiarella P., Fresegna A.M., Ciervo A., Del Frate V., Ursini C.L. (2023). Metal Oxide Nanoparticles and Graphene-Based Nanomaterials. Impact of Engineered Nanomaterials in Genomics and Epigenomics.

[80] Parvin N., Nallapureddy R.R., Mandal T.K., Joo S.W. (2022). Construction of Bimetallic Hybrid Multishell Hollow Spheres via Sequential Template Approach for Less Cytotoxic Antimicrobial Effect. IEEE Trans. Nanobiosci..

[81] Ji H., Sun H., Qu X. (2016). Antibacterial Applications of Graphene-Based Nanomaterials: Recent Achievements and Challenges. Adv. Drug Deliv. Rev..

[82] Mandal T.K. (2024). Nanomaterial-Enhanced Hybrid Disinfection: A Solution to Combat Multidrug-Resistant Bacteria and Antibiotic Resistance Genes in Wastewater. Nanomaterials.

[83] Gnanasekar S., Kasi G., He X., Zhang K., Xu L., Kang E.-T. (2023). Recent Advances in Engineered Polymeric Materials for Efficient Photodynamic Inactivation of Bacterial Pathogens. Bioact. Mater..

[84] Alfei S., Schito A.M. (2020). From Nanobiotechnology, Positively Charged Biomimetic Dendrimers as Novel Antibacterial Agents: A Review. Nanomaterials.

[85] Jiang G., Wu R., Liu S., Yu T., Ren Y., Busscher H.J., van der Mei H.C., Liu J. (2024). Ciprofloxacin-Loaded, PH-Responsive PAMAM-Megamers Functionalized with S-Nitrosylated Hyaluronic Acid Support Infected Wound Healing in Mice without Inducing Antibiotic Resistance. Adv. Healthc. Mater..

[86] Naik G., Alias R.R., Roy A.A., Mutalik S., Dhas N. (2024). Unleashing the Power of Polymeric Nanoparticles—Creative Triumph against Antibiotic Resistance: A Review. Int. J. Biol. Macromol..

[87] Panda G., Dash S., Sahu S.K. (2022). Harnessing the Role of Bacterial Plasma Membrane Modifications for the Development of Sustainable Membranotropic Phytotherapeutics. Membranes.

[88] Alfei S., Schito A.M. (2020). Positively Charged Polymers as Promising Devices against Multidrug Resistant Gram-Negative Bacteria: A Review. Polymers.

[89] Dakal T.C., Kumar A., Majumdar R.S., Yadav V. (2016). Mechanistic Basis of Antimicrobial Actions of Silver Nanoparticles. Front. Microbiol..

[90] Speranza G. (2021). Carbon Nanomaterials: Synthesis, Functionalization and Sensing Applications. Nanomaterials.

[91] Mutalik S.P., Pandey A., Mutalik S. (2020). Nanoarchitectronics: A Versatile Tool for Deciphering Nanoparticle Interaction with Cellular Proteins, Nucleic Acids and Phospholipids at Biological Interfaces. Int. J. Biol. Macromol..

[92] Gonzalez Gomez A., Hosseinidoust Z. (2020). Liposomes for Antibiotic Encapsulation and Delivery. ACS Infect. Dis..

[93] Parvin N., Mandal T.K., Joo S.-W. (2024). The Impact of COVID-19 on RNA Therapeutics: A Surge in Lipid Nanoparticles and Alternative Delivery Systems. Pharmaceutics.

[94] Bhattacharjee R., Negi A., Bhattacharya B., Dey T., Mitra P., Preetam S., Kumar L., Kar S., Das S.S., Iqbal D. (2023). Nanotheranostics to Target Antibiotic-Resistant Bacteria: Strategies and Applications. OpenNano.

[95] Zeb A., Gul M., Nguyen T.-T.-L., Maeng H.-J. (2022). Controlled Release and Targeted Drug Delivery with Poly(Lactic-Co-Glycolic Acid) Nanoparticles: Reviewing Two Decades of Research. J. Pharm. Investig..

[96] Birk S.E., Boisen A., Nielsen L.H. (2021). Polymeric Nano- and Microparticulate Drug Delivery Systems for Treatment of Biofilms. Adv. Drug Deliv. Rev..

[97] Karnwal A., Sharma V., Kumar G., Jassim A.Y., Dohroo A., Sivanesan I. (2024). Transforming Medicine with Nanobiotechnology: Nanocarriers and Their Biomedical Applications. Pharmaceutics.

[98] Santos R.S., Figueiredo C., Azevedo N.F., Braeckmans K., De Smedt S.C. (2018). Nanomaterials and Molecular Transporters to Overcome the Bacterial Envelope Barrier: Towards Advanced Delivery of Antibiotics. Adv. Drug Deliv. Rev..

[99] Parvin N., Mandal T.K., Nagajyothi P.C., Reddy P.M., Reddy N.R., Joo S.W. (2021). Highly Fluorescent Doped Fe_3_O_4_@C Nanoparticles Cross the Blood–Brain Barrier: Help in Brain Imaging and Blocking the Life Cycle of Mosquitoes. J. Clust. Sci..

[100] Li B., Mao J., Wu J., Mao K., Jia Y., Chen F., Liu J. (2024). Nano–Bio Interactions: Biofilm-Targeted Antibacterial Nanomaterials. Small.

[101] Chong K.J., Feng H., Letchumanan V., Arip M., Fatokun O., Mochamad L., Ng C.T., Chinnapan S., Selvaraja M. (2024). Tackling Microbial Resistance and Emerging Pathogens with Next-Generation Antibiotics. Prog. Microbes Mol. Biol..

[102] Rao H., Choo S., Rajeswari Mahalingam S.R., Adisuri D.S., Madhavan P., Akim A.M., Chong P.P. (2021). Approaches for Mitigating Microbial Biofilm-Related Drug Resistance: A Focus on Micro- and Nanotechnologies. Molecules.

[103] Arnold T.M., Forrest G.N., Messmer K.J. (2007). Polymyxin Antibiotics for Gram-Negative Infections. Am. J. Health Pharm..

[104] Alotaibi A.M., Alsaleh N.B., Aljasham A.T., Tawfik E.A., Almutairi M.M., Assiri M.A., Alkholief M., Almutairi M.M. (2022). Silver Nanoparticle-Based Combinations with Antimicrobial Agents against Antimicrobial-Resistant Clinical Isolates. Antibiotics.

[105] Baker A., Syed A., Alyousef A.A., Arshad M., Alqasim A., Khalid M., Khan M.S. (2020). Sericin-Functionalized GNPs Potentiate the Synergistic Effect of Levofloxacin and Balofloxacin against MDR Bacteria. Microb. Pathog..

[106] Nejabatdoust A., Mirmiran S.D., Salehzadeh A., Masouleh F.R. (2024). Effective Release of Ciprofloxacin and Rifampicin Antibiotics from Alginate-Chitosan Complex and Its Application Against Clinical Strains of Staphylococcus Aureus. Bionanoscience.

[107] Canaparo R., Foglietta F., Limongi T., Serpe L. (2020). Biomedical Applications of Reactive Oxygen Species Generation by Metal Nanoparticles. Materials.

[108] Barua S., Mitragotri S. (2014). Challenges Associated with Penetration of Nanoparticles across Cell and Tissue Barriers: A Review of Current Status and Future Prospects. Nano Today.

[109] Hadidi N., Mohebbi M. (2022). Anti-Infective and Toxicity Properties of Carbon Based Materials: Graphene and Functionalized Carbon Nanotubes. Microorganisms.

[110] Xiong M.-H., Bao Y., Yang X.-Z., Zhu Y.-H., Wang J. (2014). Delivery of Antibiotics with Polymeric Particles. Adv. Drug Deliv. Rev..

[111] Wang T., Rong F., Tang Y., Li M., Feng T., Zhou Q., Li P., Huang W. (2021). Targeted Polymer-Based Antibiotic Delivery System: A Promising Option for Treating Bacterial Infections via Macromolecular Approaches. Prog. Polym. Sci..

[112] Altun E., Aydogdu M.O., Chung E., Ren G., Homer-Vanniasinkam S., Edirisinghe M. (2021). Metal-Based Nanoparticles for Combating Antibiotic Resistance. Appl. Phys. Rev..

[113] Qu Y., Zou Y., Wang G., Zhang Y., Yu Q. (2024). Disruption of Communication: Recent Advances in Antibiofilm Materials with Anti-Quorum Sensing Properties. ACS Appl. Mater. Interfaces.

[114] Dey N., Kamatchi C., Vickram A.S., Anbarasu K., Thanigaivel S., Palanivelu J., Pugazhendhi A., Ponnusamy V.K. (2022). Role of Nanomaterials in Deactivating Multiple Drug Resistance Efflux Pumps—A Review. Environ. Res..

[115] Fernández M., Orozco J. (2021). Advances in Functionalized Photosensitive Polymeric Nanocarriers. Polymers.

[116] Mukherjee S., Mukherjee S., Abourehab M.A.S., Sahebkar A., Kesharwani P. (2022). Exploring Dendrimer-Based Drug Delivery Systems and Their Potential Applications in Cancer Immunotherapy. Eur. Polym. J..

[117] Salama B., Alzahrani K.J., Alghamdi K.S., Al-Amer O., Hassan K.E., Elhefny M.A., Albarakati A.J.A., Alharthi F., Althagafi H.A., Al Sberi H. (2023). Silver Nanoparticles Enhance Oxidative Stress, Inflammation, and Apoptosis in Liver and Kidney Tissues: Potential Protective Role of Thymoquinone. Biol. Trace Elem. Res..

[118] Yuan X., Zhang X., Sun L., Wei Y., Wei X. (2019). Cellular Toxicity and Immunological Effects of Carbon-Based Nanomaterials. Part. Fibre Toxicol..

[119] Hu X., Li D., Gao Y., Mu L., Zhou Q. (2016). Knowledge Gaps between Nanotoxicological Research and Nanomaterial Safety. Environ. Int..

[120] Reddy L.H., Arias J.L., Nicolas J., Couvreur P. (2012). Magnetic Nanoparticles: Design and Characterization, Toxicity and Biocompatibility, Pharmaceutical and Biomedical Applications. Chem. Rev..

[121] Chandler M., Panigaj M., Rolband L.A., Afonin K.A. (2020). Challenges in Optimizing RNA Nanostructures for Large-Scale Production and Controlled Therapeutic Properties. Nanomedicine.

[122] Benković M., Valinger D., Jurina T., Gajdoš Kljusurić J., Jurinjak Tušek A. (2023). Biocatalysis as a Green Approach for Synthesis of Iron Nanoparticles—Batch and Microflow Process Comparison. Catalysts.

[123] Hristozov D.R., Gottardo S., Critto A., Marcomini A. (2012). Risk Assessment of Engineered Nanomaterials: A Review of Available Data and Approaches from a Regulatory Perspective. Nanotoxicology.

[124] Lai R.W.S., Yeung K.W.Y., Yung M.M.N., Djurišić A.B., Giesy J.P., Leung K.M.Y. (2018). Regulation of Engineered Nanomaterials: Current Challenges, Insights and Future Directions. Environ. Sci. Pollut. Res..

[125] Fatima F., Siddiqui S., Khan W.A. (2021). Nanoparticles as Novel Emerging Therapeutic Antibacterial Agents in the Antibiotics Resistant Era. Biol. Trace Elem. Res..

[126] Das B., Dash S.K., Mandal D., Ghosh T., Chattopadhyay S., Tripathy S., Das S., Dey S.K., Das D., Roy S. (2017). Green Synthesized Silver Nanoparticles Destroy Multidrug Resistant Bacteria via Reactive Oxygen Species Mediated Membrane Damage. Arab. J. Chem..

[127] Srinivasarao M., Low P.S. (2017). Ligand-Targeted Drug Delivery. Chem. Rev..

[128] Yang N., Sun M., Wang H., Hu D., Zhang A., Khan S., Chen Z., Chen D., Xie S. (2024). Progress of Stimulus Responsive Nanosystems for Targeting Treatment of Bacterial Infectious Diseases. Adv. Colloid Interface Sci..

[129] Badoni A., Prakash J. (2024). Noble Metal Nanoparticles and Graphene Oxide Based Hybrid Nanostructures for Antibacterial Applications: Recent Advances, Synergistic Antibacterial Activities, and Mechanistic Approaches. Micro Nano Eng..

[130] Park W., Shin H., Choi B., Rhim W.-K., Na K., Keun Han D. (2020). Advanced Hybrid Nanomaterials for Biomedical Applications. Prog. Mater. Sci..

[131] Barman S., Kurnaz L.B., Leighton R., Hossain M.W., Decho A.W., Tang C. (2024). Intrinsic Antimicrobial Resistance: Molecular Biomaterials to Combat Microbial Biofilms and Bacterial Persisters. Biomaterials.

[132] Van Giau V., An S.S.A., Hulme J. (2019). Recent Advances in the Treatment of Pathogenic Infections Using Antibiotics and Nano-Drug Delivery Vehicles. Drug Des. Dev. Ther..

[133] Yetisgin A.A., Cetinel S., Zuvin M., Kosar A., Kutlu O. (2020). Therapeutic Nanoparticles and Their Targeted Delivery Applications. Molecules.

[134] Mitchell M.J., Billingsley M.M., Haley R.M., Wechsler M.E., Peppas N.A., Langer R. (2021). Engineering Precision Nanoparticles for Drug Delivery. Nat. Rev. Drug Discov..

[135] Hetta H.F., Ramadan Y.N., Al-Harbi A.I., Ahmed E.A., Battah B., Abd Ellah N.H., Zanetti S., Donadu M.G. (2023). Nanotechnology as a Promising Approach to Combat Multidrug Resistant Bacteria: A Comprehensive Review and Future Perspectives. Biomedicines.

[136] Smith B.R., Gambhir S.S. (2017). Nanomaterials for In Vivo Imaging. Chem. Rev..

[137] Justo-Hanani R., Dayan T. (2015). European Risk Governance of Nanotechnology: Explaining the Emerging Regulatory Policy. Res. Policy.

[138] Makabenta J.M.V., Nabawy A., Li C.-H., Schmidt-Malan S., Patel R., Rotello V.M. (2021). Nanomaterial-Based Therapeutics for Antibiotic-Resistant Bacterial Infections. Nat. Rev. Microbiol..

[139] Gupta A., Mumtaz S., Li C.-H., Hussain I., Rotello V.M. (2019). Combatting Antibiotic-Resistant Bacteria Using Nanomaterials. Chem. Soc. Rev..

[140] Karnwal A., Kumar G., Pant G., Hossain K., Ahmad A., Alshammari M.B. (2023). Perspectives on Usage of Functional Nanomaterials in Antimicrobial Therapy for Antibiotic-Resistant Bacterial Infections. ACS Omega.

[141] Xu Z., Zhao D., Lu J., Liu J., Dao G., Chen B., Huang B., Pan X. (2023). Multiple Roles of Nanomaterials along with Their Based Nanotechnologies in the Elimination and Dissemination of Antibiotic Resistance. Chem. Eng. J..

